# Endocytosis‐independent cytosolic entry of messenger RNA via fluorous bilayer zippering attenuating Toll‐like receptor signaling and enables ischemic tissue salvage

**DOI:** 10.1002/smo2.70085

**Published:** 2026-07-21

**Authors:** Yue Wang, Haitao Xie, Guoqing Xiang, Yanhua Li, Changgui Tong, Hongyan Cui, Qixian Chen, Haidong Li, Yan Zhao

**Affiliations:** ^1^ Department of Gastric Surgery Cancer Hospital of Dalian University of Technology (Liaoning Cancer Hospital & Institute) Shenyang China; ^2^ Provincial Key Laboratory of Interdisciplinary Medical Engineering for Gastrointestinal Carcinoma Cancer Hospital of Dalian University of Technology (Liaoning Cancer Hospital & Institute) Shenyang China; ^3^ Immunotherapy & Tumor Metabolism‐Microecology Research Unit Cancer Hospital of Dalian University of Technology (Liaoning Cancer Hospital & Institute) Shenyang China; ^4^ Department of Gastric Surgery Cancer Hospital of China Medical University Shenyang China; ^5^ Department of International Medical Services Cancer Hospital of Dalian University of Technology (Liaoning Cancer Hospital & Institute) Shenyang China; ^6^ Department of Endoscopy Cancer Hospital of Dalian University of Technology (Liaoning Cancer Hospital & Institute) Shenyang China; ^7^ The Second Affiliated Hospital of Dalian Medical University Dalian China; ^8^ Innovation Center of Yangtze River Delta Zhejiang University Jiaxing China; ^9^ MOE Key Laboratory of Bio‐Intelligent Manufacturing School of Bioengineering Dalian University of Technology Dalian China

**Keywords:** bio‐orthogonal click chemistry, cytosolic release, disulfide cleavage, mRNA delivery, non‐endocytic uptake, perfluorinated PEI

## Abstract

A fundamental constraint of conventional messenger RNA (mRNA) delivery systems is their obligatory trafficking through endosomal–lysosomal compartments, wherein cargo degradation and activation of endosomal Toll‐like receptors precipitate substantial translational attrition and deleterious inflammatory cascades. We herein report a chemically engineered platform that circumvents these limitations ab initio. Through strategic perfluoro‐acylation of branched polyethyleneimine (PEI, 25 kDa) with pentafluoropropionic anhydride, we install approximately 26 fluoro‐amide “zipper” moieties per polymer chain that orchestrate direct, energy‐independent trans‐bilayer translocation without recruitment of clathrin, caveolae, or lipid raft microdomains—thereby precluding lysosomal entrapment and catabolism. Bio‐orthogonal copper‐free click chemistry between azide‐ and dibenzocyclooctyne (DBCO)‐terminated PEI‐F derivatives, coupled with redox‐labile disulfide crosslinkers, engenders polyplexes of exceptional extracellular stability that undergo quantitative glutathione‐triggered disassembly within the cytosolic milieu. This endosome‐evasive entry mechanism effectively sequesters single‐stranded mRNA from Toll‐like receptor 3, TLR7, and TLR8 surveillance, establishing a “TLR‐attenuated” delivery paradigm characterized by undetectable interferon‐α, interferon‐β, TNF‐α, and IL‐6 induction. In human umbilical vein endothelial cells, GFP‐mRNA transfection exceeds 90% fluorescent positivity with 4.8‐fold superior luciferase expression relative to Lipofectamine™ 3000, whilst maintaining >95% viability. Therapeutic translatability is demonstrated in a murine hindlimb ischemia model, wherein a single 10 μg intramuscular dose of mVEGF‐A polyplexes restores blood perfusion to 118% of baseline within 28 days—representing marked superiority over the commercial gold standard and effectuating complete tissue salvage without necrosis. Comprehensive hematological and immunological profiling corroborates the absence of hematotoxicity, systemic inflammation, or innate immune activation. This modular, purely synthetic platform resolves the classical stability–availability paradox whilst eliminating the immunogenic liabilities inherent to endocytic delivery, furnishing a readily translatable scaffold for precision regenerative medicine.

## Graphical abstract

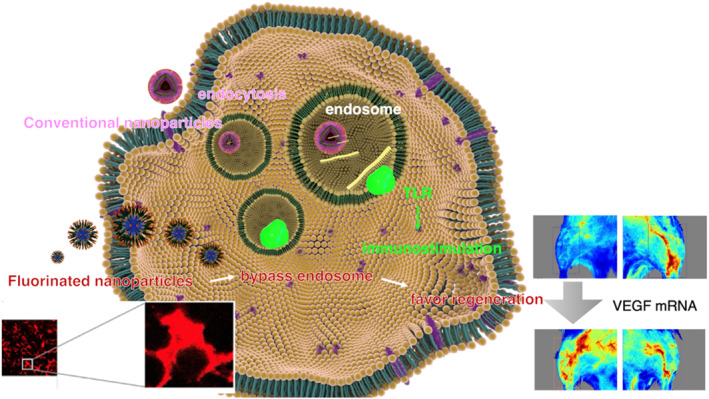

## 1 INTRODUCTION

The renaissance of messenger RNA (mRNA) therapeutics has precipitated a paradigm shift in drug development and vaccinology, offering programmable, transient gene expression with unprecedented temporal control [1, 2, 3]. Central to this translational momentum is the rational engineering of delivery vehicles capable of navigating the formidable biological barriers that impede cytosolic access—most notably the plasma membrane, extracellular nucleases, and endolysosomal entrapment [4, 5, 6, 7, 8]. While polymeric nanoparticles have emerged as versatile mRNA encapsulation platforms [9, 10, 11], virtually all extant systems remain tethered to receptor‐mediated endocytosis as their obligatory portal of entry [12]. This endocytic dependency engenders a catastrophic attrition cascade: over 90% of internalized cargo is sequestered within lysosomal compartments and subjected to acid hydrolase‐mediated degradation, while the minuscule fraction that achieves endosomal escape frequently exhibits 5′‐cap excision, phosphodiester backbone fragmentation, and translational incapacitation [13, 14]. Concomitantly, endosomal transit exposes single‐stranded mRNA to luminal surveillance by Toll‐like receptor (TLR) 3 (TLR3), TLR7, and TLR8, triggering MyD88‐and TRIF‐dependent signaling that culminates in type‐I interferon secretion, NF‐κB activation, and establishment of a pro‐inflammatory microenvironment antithetical to regenerative processes [15, 16]. A delivery architecture capable of (i) executing direct, energy‐independent plasma membrane translocation without molecular recognition of clathrin, caveolin‐1, or lipid raft constituents; (ii) reduced engagement of endosomal pattern‐recognition receptors due to bypass of the endolysosomal compartment; and (iii) executing stimulus‐responsive disassembly contingent upon cytosolic arrival would thus constitute a generational inflection point for nucleic acid therapeutics.

Peripheral arterial disease (PAD) and its terminal manifestation, chronic limb‐threatening ischemia (CLTI), crystallize the clinical imperative for such innovation. With a global prevalence exceeding 200 million and >1.5 million major amputations annually [17, 18], CLTI represents a burgeoning epidemic for which current revascularization modalities—surgical bypass or endovascular recanalization—are anatomically feasible in merely 25%–30% of patients [19, 20]. The remainder, designated “no‐option” CLTI, progress inexorably toward tissue necrosis despite maximal medical therapy. Vascular endothelial growth factor‐A (VEGF‐A) remains the most biologically validated pro‐angiogenic stimulus; however, 3 decades of clinical investigation employing recombinant protein formulations or viral gene therapy have yielded, at best, marginal efficacy, attributable fundamentally to the inability to achieve sustained, spatially restricted expression without systemic dissemination and dose‐limiting edema [21]. Messenger RNA therapeutics theoretically circumvent these pharmacokinetic constraints through pulsatile, self‐limited protein synthesis, yet conventional lipid nanoparticles exhibit rapid hepato‐splenic sequestration, negligible skeletal muscle retention, and dose‐proportional activation of innate immune sensors that compromise both safety and therapeutic index [22, 23]. The unmet need—‐and the central objective of this investigation—‐is a delivery platform capable of confining robust VEGF‐A translation to the ischemic microenvironment, abrogating pattern‐recognition receptor signaling, and achieving these outcomes from microgram‐scale doses amenable to office‐based intramuscular administration.

Polyethyleneimine (PEI) has remained the gold standard among polycationic nucleic acid delivery vehicles for over 2 decades, attributable to its exceptional proton sponge effect and unparalleled transfection efficiency across diverse cell types [24]. Branched PEI (25 kDa) achieves superior transfection compared to virtually all synthetic alternatives, yet its clinical translation has been severely constrained by dose‐limiting cytotoxicity, rapid renal clearance, and non‐degradable backbone accumulation—liabilities that precipitate substantial inflammatory responses and preclude repeated administration [25]. Consequently, despite extensive preclinical investigations, no native PEI formulation has achieved regulatory approval for human use.

Recent translational efforts have therefore focused on PEI engineering strategies to mitigate these inherent deficiencies. Notable progress includes: (i) PEGylation to improve pharmacokinetic profiles and reduce immunogenicity [26], exemplified by in vivo jetPEI® (Polyplus‐transfection®) which has entered clinical trials for DNA vaccination and siRNA delivery [27]; (ii) biodegradable linkages such as ester, amide, or disulfide bonds integrated into the PEI backbone or side chains to enable metabolic clearance with disulfide‐crosslinked PEI nanoparticles for CRISPR delivery [28]; (iii) targeted modifications with tumor‐homing ligands (RGD, folate, transferrin) to enhance tissue specificity [29]; and (iv) hybrid systems combining PEI with lipids, cyclodextrins, or inorganic nanoparticles to leverage complementary delivery mechanisms [30]. While these approaches have incrementally improved the therapeutic index, none have fundamentally resolved the central paradox of PEI‐based delivery: the obligatory trafficking through endosomal compartments that simultaneously degrades cargo, activates innate immune surveillance, and necessitates high carrier doses that exacerbate toxicity.

We herein posit that a chemically programmable PEI nanoconstruct—perfluoro‐acylated for membrane‐active “zippering,” bio‐orthogonally cross‐linked for extracellular stabilization, and disulfide‐bridged for glutathione (GSH)‐triggered cytosolic disassembly—can satisfy these stringent design criteria.

Our PEI‐F(N_3_&DBCO) architecture represents a departure from these incremental modifications by implementing three orthogonal, mechanism‐driven chemistries that collectively transform PEI from a conventional polycation into a genuinely “smart” delivery vehicle: (1) Perfluoro‐acylation for membrane zippering: Rather than merely modulating hydrophilicity or reducing toxicity, perfluoroalkyl installation fundamentally reconfigures PEI's membrane interaction mechanism—from receptor‐mediated endocytosis to direct, energy‐independent trans‐bilayer translocation. This fluorous “zippering” effect constitutes a mechanistic paradigm shift that eliminates the endosomal bottleneck entirely, rather than merely enhancing endosomal escape efficiency. (2) cycloaddition introduces programmable extracellular durability without compromising intracellular bioavailability. Unlike conventional PEGylation or surface charge masking, this chemistry enables precise, stimulus‐responsive disassembly contingent upon cytosolic arrival—a true “locked‐outside, labile‐inside” behavior unattainable with passive formulations. (3) Disulfide cleavage for glutathione‐gated release: The redox‐labile hinge functions as a molecular sensor that detects the ∼10^3^‐fold glutathione gradient between extracellular and intracellular environments [31], executing quantitative payload liberation within minutes of membrane traversal. This responsive element transforms the carrier from a passive container into an active, environment‐sensing delivery system.

Collectively, the functionalization elevates PEI beyond its historical role as a “dumb” but efficient polycation into an intelligent, multi‐responsive nanoconstruct capable of autonomous decision‐making at each stage of the delivery trajectory: membrane recognition and penetration, extracellular stabilization, intracellular sensing, and triggered payload release. This “smart molecule” approach directly addresses the three classical PEI limitations—cytotoxicity, immunogenicity, and endosomal entrapment—through mechanism‐driven design rather than empirical modification, positioning our platform for clinical translation where prior PEI iterations have stalled.

**SCHEME 1:**
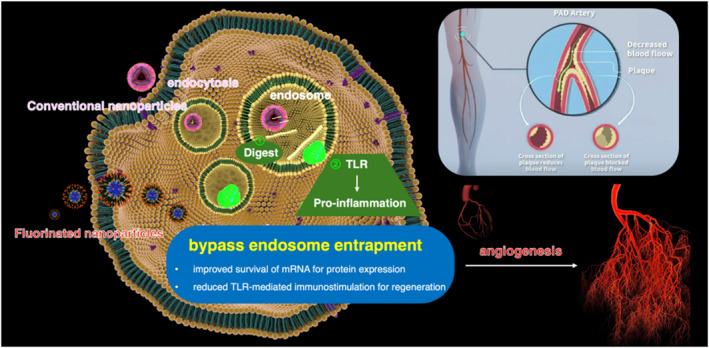
Schematic illustration of the fluorinated messenger RNA delivery constructs that spontaneously traverse the plasma membrane via an endocytosis‐independent pathway, bypass endosomal entrapment and degradation, and substantially evade endosomal TLR‐mediated immunostimulation, thereby enabling robust cytosolic mRNA expression for potential neo‐vasculature development in treatment of peripheral arterial disease. mRNA, messenger RNA.

By circumventing endosomal compartments entirely, we anticipate sequestering nucleic acid cargo from TLR3/7/8 signaling, thereby attenuating TLR‐mediated inflammatory responses compared to conventional endocytotic carriers that maximizes regenerative bioactivity whilst minimizing inflammatory entropy (Scheme 1). The modular, purely synthetic nature of this architecture portends immediate extensibility to diverse therapeutic payloads—including alternative angiogenic growth factors, immunomodulatory cytokines, and CRISPR‐Cas9–encoding mRNA—positioning this chemistry‐driven approach as a foundational scaffold for precision regenerative medicine.

## 2 MATERIALS AND METHODS

### 2.1 Materials

Branched PEI (bPEI, *M*_w_ 25 kDa) was purchased from Sigma‐Aldrich (St Louis, MO) and dried in vacuo (40°C, <0.1 mbar, 12 h) prior to use. Pentafluoropropionic anhydride (PFPA, ≥98%) was obtained from Fluorochem (Hadfield, UK) and stored under argon at −20°C. DBCO‐ss‐COOH (4‐(11,12‐didehydrodibenzo[b,f]azocin‐5(6H)‐yl)‐4‐oxo‐3‐oxapentanoic acid, ≥95% HPLC) were supplied by Click Chemistry Tools (Scottsdale, AZ) and BroadPharm (San Diego, CA), respectively, and used without further purification. NHS‐PEG_4_‐N_3_ (azido‐PEG_4_‐NHS ester, ≥95% HPLC) was purchased from SinoPEG Co. Ltd (Xiamen, China). 1‐Ethyl‐3‐(3‐dimethylaminopropyl)carbodiimide hydrochloride (EDC·HCl, ≥99%) and N‐hydroxysuccinimide (NHS, ≥98%) were from GL Biochem (Shanghai, China) and Sigma‐Aldrich, respectively. Anhydrous *N*, *N*‐dimethylformamide (DMF, CHROMASOLV™ Plus, ≤0.005% H_2_O) and anhydrous methanol (CHROMASOLV™ Plus, ≤0.005% H_2_O) were purchased from Merck (Darmstadt, DE). All solvents were de‐gassed by three freeze–pump–thaw cycles and stored over activated 3 Å molecular sieves under argon. Deionised water (18.2 MΩ cm, 25°C) was obtained from a Milli‐Q® Advantage A10 system (Merck Millipore, Burlington, MA, USA). The HUVEC cell line was obtained from the American Type Culture Collection (Manassas, VA). Cells were maintained in high‐glucose Dulbecco's Modified Eagle Medium (DMEM, Gibco) supplemented with VEGF, 10% (v/v) fetal bovine serum (FBS, Gibco, South America origin) and 1% (v/v) penicillin–streptomycin (10,000 U mL^−1^, Gibco). All media were tested for endotoxin levels ≤0.1 EU mL^−1^ (Limulus amebocyte lysate assay, Charles River). Cells were cultured at 37°C in a humidified atmosphere containing 5% CO_2_ and 95% air. MTT (3‐(4,5‐dimethylthiazol‐2‐yl)‐2,5‐diphenyltetrazolium bromide) and Triton X‐100 were from Sigma‐Aldrich. DAPI (4′,6‐diamidino‐2‐phenylindole) and ProLong™ Diamond Antifade Mountant were obtained from Invitrogen. 0.22 μm PVDF and PES syringe filters were from Millipore. Firfly mLUC mRNA was purchased from Jinlin Biotech. Co.

### 2.2 Synthesis of PEI‐F (perfluoro‐acylated bPEI)

As shown in Supporting Information S1; Figure S1, branched polyethylenimine (bPEI, *M*_w_ 25 kDa, Sigma‐Aldrich, ≥99%) was dried under vacuum at 40°C for 12 h to remove residual moisture, and then dissolved in anhydrous methanol (CH_3_OH, Merck, CHROMASOLV™ Plus, ≤0.005% H_2_O) at a concentration of 10 mg mL^−1^ (tolerable window 2–20 mg mL^−1^) under an inert N_2_ atmosphere in a septum‐sealed round‐bottom flask. Pentafluoropropionic anhydride (PFPA, Fluorochem, ≥98%) was introduced dropwise (addition rate 0.5 mL min^−1^) via a gas‐tight syringe under magnetic stirring (500 rpm) at a molar ratio of 1:120 (bPEI repeat unit: PFPA; acceptable range 1:80–1:160). The reaction vessel was immersed in a thermostated oil bath (±0.1°C) and maintained at 25–30°C for 24–48 h under N_2_. Upon completion, the reaction medium was quenched by addition of 2 volumes of ice‐cold 0.01 M HCl and immediately transferred into pre‐wetted regenerated‐cellulose dialysis tubing (MWCO 3.5 kDa, Spectrum; acceptable 3–5 kDa) that had been pre‐treated with EDTA (1 mM) and NaHCO_3_ (2%) to remove trace metal ions. Dialysis was performed sequentially against 0.01 M HCl (3 × 12 h, 200‐fold volume exchange each) and ultrapure water (3 × 12 h, 18.2 MΩ cm at 25°C) for a cumulative 36 h (flexible 18–72 h). The retentate was filtered through a 0.22 μm PVDF syringe filter, flash‐frozen in liquid N_2_, and lyophilized (Christ Alpha 2‐4 LSC, −80°C condenser, 0.01 mbar) for ≥48 h to yield PEI‐F, hereafter designated PF, as a white, electrostatically fluffy solid. In reference to PEI (Supporting Information S1; Figure S2), degree of substitution (DS) was quantified by ^1^H–NMR using trifluoroacetic acid as the internal standard (Supporting Information S1; Figure S3).

### 2.3 Synthesis of PEI‐F(DBCO)

DBCO‐ss‐COOH (42 μmol, 1.00 equiv; Click Chemistry Tools, ≥95% HPLC) was dissolved in anhydrous DMF (Acros, 99.8%, ≤0.005% H_2_O) that had been sparged with dry N_2_ for 30 min 1‐Ethyl‐3‐(3‐dimethylaminopropyl)carbodiimide hydrochloride (EDC·HCl, 54.6 μmol, 1.30 equiv, GL Biochem, ≥99%) and N‐hydroxysuccinimide (NHS, 54.6 μmol, 1.30 equiv, Sigma‐Aldrich, ≥98%) were added in quick succession, and the faint‐yellow solution was stirred (600 rpm) at 25–30°C under N_2_ for 3–5 h to generate the NHS‐ester in situ. Activation efficiency was verified by RP‐HPLC (C18, 5 μm, 4.6 × 250 mm; mobile phase A: 0.1% TFA in H_2_O, B: 0.1% TFA in MeCN; gradient 5%–95% B over 20 min; λ = 309 nm) showing ≥95% consumption of free acid. The activated ester solution was cooled to 0°C and added dropwise (0.2 mL min^−1^) to a methanolic PF solution (4.6 mg mL^−1^, acceptable 4–5 mg mL^−1^) at a DBCO‐ss‐COOH: PF molar ratio of 8:1. The mixture was allowed to warm to 25–30°C and stirred for 12–24 h under N_2_. Progress was monitored by SEC‐MALS (Shimadzu LC‐20AD, Wyatt DAWN HELEOS‐II) to confirm the quantitative shift of molecular weight distribution. The crude conjugate was diluted 1:1 (v/v) with 0.01 M HCl and dialysed (MWCO 3.5 kDa) against 0.01 M HCl (3 × 12 h, 200‐fold excess) and ultrapure water (3 × 12 h) as described in 2.1 section. The retentate was filtered (0.22 μm), flash‐frozen, and lyophilized to afford PF‐DBCO as a pale‐yellow, friable solid. DBCO loading density was determined by UV‐vis spectroscopy (*ε*_309_ = 12,000 M^−1^ cm^−1^ in DMF) and corroborated by ^1^H NMR integration (Supporting Information S1; Figure S4).

### 2.4 Synthesis of PEI‐F(N3)

NHS‐PEG_4_‐N_3_ (8.0 equiv, BroadPharm, ≥95% HPLC) was dissolved in anhydrous CH_3_OH (≤0.005% H_2_O) and added to a methanolic PF solution (4.5 mg mL^−1^, acceptable 4–5 mg mL^−1^) under N_2_. The reaction was stirred (500 rpm) at 25–30°C for 4–12 h, protected from light. The consumption of NHS‐ester was verified by RP‐HPLC (C18, λ = 260 nm) showing ≥97% conversion. The solution was acidified to pH 3.0 with 0.01 M HCl and subjected to dialysis (MWCO 3.5 kDa) against 0.01 M HCl (3 × 12 h) and ultrapure water (3 × 12 h) as above. The purified PF‐N_3_ solution was either used immediately for click reactions or aliquoted (1 mL), snap‐frozen in liquid N_2_, and lyophilized for long‐term storage (−80°C, desiccated). Azide density was quantified by a modified Ellman's assay after Staudinger reduction and verified by ^1^H NMR (PEG_4_ –CH_2_– signal at 3.65 ppm, Supporting Information S1; Figure S5).

### 2.5 Construction of VEGF mRNA

The VEGF‐A165 coding sequence was cloned into pUC57‐Kan (GenScript) downstream of the T7 promoter. For in vitro transcription, the plasmid was linearized at the 3′ end of the insert using XbaI (New England Biolabs) purified by phenol‐chloroform extraction followed by ethanol precipitation, and transcribed using Hi‐T7 RNA polymerase (New England Biolabs) according to the manufacturer's instructions. The complete plasmid sequence file is provided as the enclosed supporting information file. Detailed information and procedures are provided in the Supporting information. Functional validation: 500 ng RNA transfected into HUVECs produced 5.2‐fold more secreted VEGF‐A_165_ than the commercial control at 24 h. Aliquots (1 μg/μL) were flash‐frozen in liquid N_2_ and stored at −80°C.

Notably, to avoid the premature degradation of mRNA, the following precautions were strictly conducted: (1) RNase‐free reagent preparation: All aqueous solutions were prepared using RNase‐free ultrapure water. (2) Controlled experimental environment: All procedures were performed in a dedicated RNase‐free workspace. Work surfaces and equipment were pre‐treated with RNase decontamination agents (e.g., RNaseZap™, Thermo Fisher) prior to use. (3) Chemical RNase inhibition: For heparin displacement and other incubation‐based assays including electrophoresis assay, 10 mM tris (2‐carboxyethyl)phosphine (TCEP) was included in the reaction buffer to inhibit RNase activity and prevent enzymatic degradation of mRNA. (4) Temperature control: Polyplex formulation and physicochemical characterization steps were performed on ice or at 2–8°C to minimize thermally induced mRNA degradation.

### 2.6 Preparation of mRNA polyplexes

PEI‐F(DBCO) and PEI‐F(N_3_) were separately dissolved in sterile, RNase‐free PBS (pH 7.4, Gibco, 10× diluted with DEPC‐treated water) at 0.33 mg mL^−1^ and filtered through 0.22 μm PES syringe filters. Equal volumes of PF‐DBCO solution, PF‐N_3_ solution and mRNA (10 μM in PBS, Dharmacon, desalted and HPLC‐purified) were combined at a volumetric ratio of 1.5:1.5:1 in low‐binding Eppendorf tubes. The mixture was gently pipetted (3×, wide‐bore tip) and incubated at 25°C for 3–5 min to permit spontaneous polyplex self‐assembly. Final mRNA concentration was adjusted to 50 nM for downstream assays unless stated otherwise. Polyplex morphology was imaged by TEM (Talos L120C, 200 mesh Cu grids, 2% uranyl acetate negative stain) within 30 min of preparation to avoid ageing artefacts.

### 2.7 Physicochemical characterizations

Hydrodynamic diameter (D_h_), polydispersity index and ζ potential of polyplexes were determined by dynamic light scattering (DLS) and laser Doppler micro‐electrophoresis using a Zetasizer Nano‐ZS (Malvern, UK) equipped with a 633 nm He‐Ne laser and avalanche photodiode detector at 173° back‐scatter angle. Samples were equilibrated at 25.0 ± 0.1°C for 120 s and measured in disposable folded‐capillary cells (DTS1070). Each acquisition consisted of 12 runs of 10 s; data were analyzed by cumulative fit (D_h_) and Smoluchowski model (ζ potential). DTS1070 cells were used for zeta potential measurements. All measurements were performed in triplicate on independently prepared batches.

### 2.8 Resistance to polyion exchange reactions with heparin

Heparin was dissolved in a stock solution of 10 mM HEPES buffer (pH 8.0) at a concentration of 10 mg ml^−1^. Then, solutions of a variety of mRNA delivery constructs (18 μL) were combined with an aliquot of heparin at a concentration. The samples underwent a polyion‐exchanging reaction for 6 h at room temperature. Reaction solutions were electrophoresed on 0.9% agarose gel with Tris‐acetate buffer (pH 7.4) as running buffer at 100 V for 60 min. The mRNA was visualized in the gel by ethidium bromide staining under UV illumination using an incident light fluorescence detector.

### 2.9 Cytotoxicity

HUVECs were maintained in complete medium at 37°C, 5% CO_2_, 95% humidity. Cells were seeded in 96‐well plates (5 × 10^3^ cells per well, 100 μL) and allowed to attach for 24 h. Culture medium was replaced with 90 μL fresh medium, followed by the addition of 10 μL mRNA‐encapsulating polyplexes. After 24 h exposure, 20 μL MTT reagent (0.5 mg mL^−1^ in PBS) was added and incubated for 4 h. Formazan crystals were solubilized with 100 μL DMSO (spectrophotometric grade) and the absorbance was recorded at 570 nm (reference 630 nm) on a microplate reader. Cell viability is expressed as Viability (%) = (A_treated_ − A_blank_)/(A_control_ − A_blank_) × 100. Each concentration was tested in octuplicate, and three independent experiments were conducted on different passages.

### 2.10 ELISA for quantification of interferon‐α production

RAW264.7 cells were incubated for 48 h in DMEM–10% FBS with the indicated mRNA formulations. Culture supernatants (100 μL/well) were harvested and assayed for IFN‐α using the Mouse IFN‐α ELISA Kit (PBL Interferon Source) according to the manufacturer's instructions. Data are mean ± SD of four independent wells.

### 2.11 Cellular uptake

Cells were seeded in 8‐well ibiTreat chamber slides (2 × 10^4^ cells per well, 300 μL) and cultured for 24 h. Medium was replaced with 250 μL Opti‐MEM™ reduced‐serum medium containing Cy5‐labelled mRNA polyplexes (0.050 mg mL^−1^). After 2–6 h of incubation (37°C, 5% CO_2_), cells were washed 3× with ice‐cold PBS, fixed with 4% paraformaldehyde (15 min, RT), permeabilized with 0.1% Triton X‐100 (5 min), and counter‐stained with DAPI (1 μg mL^−1^, 10 min). Slides were mounted with ProLong™ Diamond antifade medium and imaged by confocal laser‐scanning microscopy (CLSM; Zeiss LSM 880, Plan‐Apochromat 63×/1.4 NA oil objective, 405 and 633 nm excitation). For flow‐cytometric quantification, cells were trypsinized, resuspended in ice‐cold PBS +2% FBS, and analyzed on a BD FACSCanto II (10,000 events per sample, Cy5 detected in APC‐A channel). Mean fluorescence intensity was normalized to untreated control.

### 2.12 Endocytosis pathway mapping

HUVECs (1 × 10^5^ per well, 24‐well format) were seeded on glass coverslips (CLSM) or suspension‐grade plates (FCM) and grown overnight to 70%–80% confluence. After a 30‐min equilibration in serum‐free DMEM, cells were pre‐treated for 30 min with one of the following inhibitors: chlorpromazine (10 μg mL^−1^, clathrin‐mediated), methyl‐β‐cyclodextrin (5 mM, lipid‐raft), genistein (200 μM, caveolae), sodium azide (0.05% w/v, ATP‐depletion), or incubated at 4°C to block energy‐dependent uptake. Cy5‐labelled mRNA (50 ng) condensed with PEI‐F(N_3_&DBCO) at N/P 10 was then added in 200 μL Opti‐MEM and allowed to associate for 1 h under the same inhibitor/temperature condition. Following three ice‐cold PBS washes, CLSM samples were fixed (4% PFA, 15 min), nuclei counter‐stained with Hoechst 33,342 and mounted in ProLong Gold. FCM samples were trypsinized, quenched with 2% FBS‐PBS, kept on ice, and filtered. Cy5 fluorescence was acquired on a Zeiss LSM 880 (≥50 cells per field, three fields per replicate) or a BD FACSymphony (100,000 single‐cell events); median fluorescence intensity was normalized to the untreated 37°C control (set as 100%) and analyzed by student *t* test.

### 2.13 mRNA expression efficiencies

HUVECs were seeded in 24‐well plates at a density of 20,000 cells for 24 h at 37°C with a CO_2_ concentration of 5%. After replacing the culture medium with fresh medium, various mRNA therapeutics, containing 1 μg Luc or GFP mRNA per well, were added to each well. Following 24 h of incubation, the medium was replaced with 400 μL of fresh medium, followed by another 24 h of incubation. Luciferase expression was evaluated using a Luciferase assay system (Promega, Madison, WI) and a GloMax^TM^ 96 microplate luminometer (Promega) following the manufacturer's protocol (*n* = 4). The protein quantity in the cell lysates was determined using a MicroBCA™ Protein Assay Reagent Kit according to the manufacturer's protocol.

### 2.14 qPCR for quantification of IFN‐β1 and IL‐8

For RNA extraction, TRIzol™ Reagent (Invitrogen) was used according to the manufacturer's protocol, involving chloroform phase separation, isopropanol precipitation, and a 75% ethanol wash; for reverse transcription, PrimeScript™ RT Master Mix (Takara) was employed with incubation at 37°C for 15 min followed by 85°C for 5 s using oligo(dT) primers; the qPCR primers were as follows—IFN‐β1 forward primer 5′‐ATGAGTGGTGGTTGCAGGC‐3′ and reverse primer 5′‐TGACCTTTCAAATGCAGTAGATTCA‐3′, IL‐8 (mouse Cxcl15) forward primer 5′‐GGTGGCATAGAGTACAAACCC‐3′ and reverse primer 5′‐GCAAGGACAACCCTTCAGGA‐3′, and Gapdh forward primer 5′‐AGGTCGGTGTGAACGGATTTG‐3′ and reverse primer 5′‐TGTAGACCATGTAGTTGAGGTCA‐3'; the cycling conditions used SYBR Green (Takara) with an initial denaturation at 95°C for 30 s, followed by 40 cycles of 95°C for 5 s and 60°C for 34 s, and concluded with a melt curve analysis.

### 2.15 Angiogenesis in peripheral ischemia animal model

The peripheral ischemia animal model was established on Balb/c mice which had undergone hindlimb ischemic surgery. The potential revascularization efficacy were evaluated by means of local dosages (intramuscular injection) of mVEGF therapeutics. Female Bal b/c mice (4 weeks old, 20 ± 2 g) were obtained from a provider and allowed a 1‐week acclimatization period in an SPF facility with controlled temperature (22 ± 2°C), humidity (50%–60%), and a 12‐h light/dark cycle. Surgical interventions were performed under isoflurane anesthesia, with postoperative analgesia provided as appropriate. Their hindlimb ischemic surgeries involved an incision being made into their skin from the medial thigh to the knee and the membranes covering their muscles being carefully dissected. Furthermore, their neurovascular bundles were exposed by piercing the membranous femoral sheath, and the external iliac artery, femoral artery, and peroneal artery, respectively, were separated. Ligations were performed in the proximal and distal femoral arteries with 8/0 PGA fast absorbing sutures and dissections were performed between these two ligations. Finally, the mice were sutured and they received intraperitoneal injections of 10% ampicillin (10 mL/kg). The treated Balb/c female mice were divided randomly into 3 groups: saline (blank, *n* = 5), mVEGF‐Lipofectamine^TM^ 3000 and mVEGF‐PEI‐F(N_3_&DBCO). Pertaining to the treatment groups, 0.1 mL solutions containing 10 μg mVEGF were locally injected into the mice. The vasculatures in their hindlimbs were monitored for up to 28 days.

### 2.16 CatWalk gait analysis

Gait analyses were conducted using the CatWalk system (Noldus, Wageningen, Netherlands), a widely recognized and validated tool for assessing locomotor function in rodents. This system allows for precise and objective measurements of various gait parameters by capturing the footprints of freely walking mice on a glass plate. The mice were acclimated to the testing environment and allowed to walk freely within an enclosed runway situated on a glass plate. The footprints were captured using a high‐resolution charge‐coupled device camera positioned beneath the runway, ensuring accurate and consistent data collection.

The captured footprints were then analyzed using the CatWalk XT 10.0 software (Noldus), which provides a comprehensive suite of tools for extracting and quantifying gait parameters. For each time point, three uninterrupted runs were recorded and analyzed to ensure robust and reliable data. The following three well‐established parameters, which are crucial for functional SCI analyses, were meticulously measured:

Maximum Hindpaw Contact Area: This parameter measures the largest area of contact made by the hindpaw with the glass plate during a single step. It provides valuable insights into the gait stability and weight distribution of the mouse, reflecting the extent of foot placement and the overall balance during locomotion. A larger contact area typically indicates a more stable and coordinated gait, while a smaller area may suggest instability or impairment.

Maximum Hindpaw Contact Intensity: This metric is defined as the mean of the 15 most intense pixel values captured during the hindpaw's contact with the glass plate. It reflects the pressure exerted by the hindpaw, providing information on the force distribution and the overall vigor of the step. Higher contact intensity values indicate a stronger and more forceful step, which is often associated with normal locomotor function. Conversely, lower values may suggest reduced strength.

Distance Between the Hindpaw and Preceding Ipsilateral Forepaw Positions: This parameter measures the spatial relationship between the hindpaw and the preceding ipsilateral forepaw during locomotion. Specifically, it quantifies the distance between the center of the hindpaw footprint and the center of the preceding ipsilateral forepaw footprint. This measurement is crucial for evaluating the coordination and stride length between the hindpaw and the forepaw, which are essential for understanding gait patterns and potential impairments.

### 2.17 Statistical analysis

The statistical significance of the results was determined using the student's *t*‐test with a two‐tailed distribution and two‐sample unequal variance, utilizing the *t*‐test function of Microsoft Excel. A *p*‐value less than 0.05 was considered statistically significant.

## 3 RESULTS AND DISCUSSION

### 3.1 Synthesis of fluorinated polycatiomers with stimuli‐responsive bio‐orthogonal click chemistry module

We herein report the rational design of a perfluorinated polyethyleneimine (PEI‐F) delivery architecture engineered to circumvent the classical endocytic bottleneck of mRNA therapeutics. Perfluoro‐acylation is hypothesized to fundamentally reconfigure the amphiphilic character of the polycationic scaffold, engendering spontaneous, energy‐independent trans‐bilayer translocation that bypasses endosomal entrapment entirely. To confer extracellular robustness without compromising intracellular bioavailability, we integrated a binary switching mechanism: azide‐functionalized PEI‐F(N_3_) and dibenzocyclooctyne (DBCO)‐terminated counterparts [PEI‐F(DBCO)] undergo strain‐promoted, copper‐free azide–alkyne cycloaddition to molecularly weld polyplexes into a stable network under oxidative, low‐glutathione extracellular conditions. The cross‐linker incorporates a reductively labile disulfide hinge that undergoes quantitative cleavage upon cytosolic entry, effectuating payload release in response to the millimolar glutathione gradient.

The synthetic trajectory of PEI‐F derivatives is schematically delineated in Figure S1. Branched PEI (bPEI, *M*_n_ 25 kDa) was subjected to regioselective perfluoro‐amidation via treatment with pentafluoropropionic anhydride (PFPA) in anhydrous methanol, affording fluorinated scaffolds designated PEI‐F. ^1^H‐NMR spectroscopic quantification (Supporting Information S1; Figure S3) revealed a DS of approximately 26.0 pentafluoropropionamide units per polymer chain; the emergence of a discrete methylene resonance at *δ* 3.50 ppm confirmed preferential acylation of secondary amines over primary terminal amines. Notably, detailed evidence was verified by two‐dimensional nuclear magnetic resonance (2D NMR) spectroscopy, specifically heteronuclear single quantum coherence. The corresponding assignment is summarized in Supporting Information S1; Table S1, confirming the proposed structure of our synthesized PEI‐F (Supporting Information S1; Figure S6).

Subsequent orthogonal functionalization employed a hetero‐bifunctional linker harboring a DBCO moiety and a redox‐cleavable disulfide tether (DBCO‐ss‐COOH). Conjugation to PEI‐F was executed via carbodiimide‐mediated amidation (EDC/NHS), with ^1^H‐NMR authentication (Supporting Information S1; Figure S4) revealing characteristic DBCO aromatic signals (*δ* 7.0–7.5 ppm) corresponding to a grafting density of approximately 7.4 units per polymer. In parallel, complementary azide installation was achieved through the reaction of PEI‐F with NHS‐PEG_4_‐N_3_ (8 equiv), yielding PEI‐F(N_3_). The diagnostic triplet at *δ* 3.65 ppm, ascribed to PEG backbone methylene protons (Supporting Information S1; Figure S5), indicated approximately 7.5 azide residues per chain—corroborating near‐quantitative amidation efficiency and stoichiometric balance between click‐reactive partners.

Notably, conventional disulfide crosslinking relies on thiol–thiol oxidation, typically requiring extended incubation (hours to days) under mildly oxidative conditions (e.g., dialysis against dilute hydrogen peroxide or ambient oxygen) to achieve sufficient crosslinking density. This protracted oxidative exposure poses substantial risks to mRNA integrity: reactive oxygen species generated during thiol oxidation can induce phosphodiester backbone scission, nucleobase oxidation (particularly guanine), and 5′‐cap damage—collectively compromising translational competence.

In contrast, strain‐promoted azide–DBCO cycloaddition proceeds with second‐order rate constants under physiological conditions without any oxidative component. Moreover, the click reaction's insensitivity to oxygen permits open‐vessel formulation under the standard laboratory conditions—a practical advantage that facilitates clinical translation. In addition, thiol‐functionalized PEI derivatives exhibit notorious storage instability, with spontaneous oxidation, disulfide scrambling, and thiol–ene adduct formation limiting shelf‐life. The azide and DBCO functionalities employed in our system are chemically inert under standard storage conditions, enabling lyophilized precursor stability.

### 3.2 Manufacture of multifunctional mRNA delivery polyplexes integrating fluorination motif and in situ reversible crosslinking function

**FIGURE 1:**
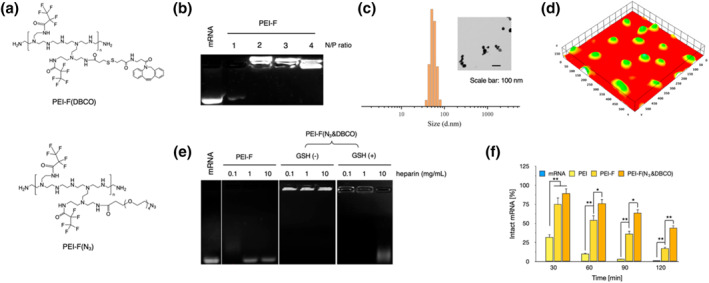
Physiochemical properties of mRNA‐encapsulating polyplexes. (a) Chemical structures of PEI‐F(N_3_) and PEI‐F(DBCO). (b) Gel electrophoresis of mRNA‐encapsulating polyplexes at varied N/P ratios. (c) dynamic light scattering (DLS) measurement for mRNA‐encapsulating polyplexes of PEI‐F(N_3_&DBCO) at an N/P ratio of 2. Microscopic morphologies of mRNA‐encapsulating polyplexes of PEI‐F(N_3_&DBCO) at N/P ratio of 2 by TEM measurement. (d) Fluid AFM measurement for insights into morphologies of mRNA‐encapsulating polyplexes of PEI‐F(N_3_&DBCO) at N/P ratio of 2. (e) Reversible colloidal stabilities in resistance to exchange reactions with polyanionic heparin at varied concentration (6 h). (f) tolerability of mRNA‐encapsulating polyplexes in 50% fetal bovine serum (FBS) (mean ± s.e.m., *n* = 4, *p < 0.05, **p < 0.01; Student's *t*‐test).

Click‐competent macromolecular precursors (Figure 1a) were co‐assembled via spontaneous polyion complexation: equimolar PEI‐F(DBCO) and PEI‐F(N_3_) solutions (0.33 mg mL^−1^ each) were combined with mRNA (0.1 mg mL^−1^) at a volumetric ratio of 1.5:1.5:1, followed by incubation (25°C, 30 min) to permit complete azide–DBCO cycloaddition. Agarose gel electrophoretic retardation confirmed quantitative mRNA complexation at N/*p* ≥ 2 (Figure 1b). To attenuate the inherent cytotoxicity of polycationic delivery constructs, an N/P ratio of 2.0 was selected for all subsequent investigations.

Physicochemical characterization revealed narrowly dispersed nanoparticles with a hydrodynamic diameter (D_h_) of 39.3 ± 2.1 nm (PDI: 0.10 ± 0.01) and ζ‐potential of +24.3 ± 1.1 mV (Table 1)—dimensions comparable to unmodified PEI or PEI‐F analogues. In contrast, the hydrodynamic diameter of the naked mRNA was determined to be approximately 76.3 ± 10.4 nm, with a zeta potential of approximately −47.3 ± 3.9 mV. The results indicate the successful complexation by PEI or PEI‐F analogues, which subsequently led to compaction of mRNA into nanoscaled delivery constructs. Transmission electron microscopy (uranyl acetate negative stain, inset Figure 1c) visualized uniformly spherical architectures with electron‐dense mRNA cores, while fluid‐phase atomic force microscopy (Figure 1d) corroborated the nanoscale topography inferred from DLS.

**TABLE 1:** Physiochemical properties of mRNA‐encapsulating polyplexes.

Polyplexes	Diameter (nm)	PDI	ζ potential (mV)
PEI	41.2 ± 1.3	0.10 ± 0.02	+23.1 ± 1.3
PEI‐F	40.3 ± 2.6	0.10 ± 0.02	+25.0 ± 1.2
PEI‐F(N_3_&DBCO)	39.3 ± 2.1	0.10 ± 0.01	+24.3 ± 1.1

**FIGURE 2:**
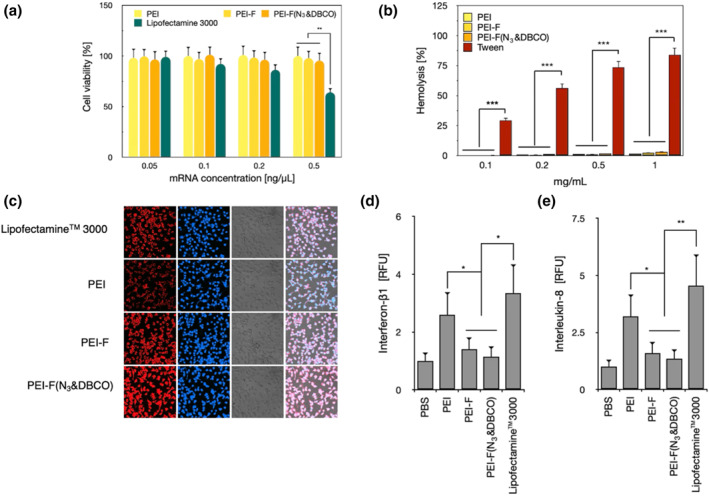
Biocompatibilities and immunogenicity of mRNA‐encapsulating polyplexes. (a) Cell viabilities of HUVECs upon 24 h incubation in presence of a variety of mRNA‐encapsulating polyplexes (mean ± s.d., *n* = 4, **p < 0.01, student *t* test). (b) Hemolytic activities of sheep red blood cells upon 2 h incubation with varied concentrated delivery materials (***p < 0.005; Student's *t*‐test, mean ± s.d., *n* = 4). (c) confocal laser scanning microscopy (CLSM) measurement for assessment of overall cellular internalization of mRNA‐encapsulating polyplexes into RAW264.7 cells. (d) Interferon‐β (IFN‐β): at 4 h post incubation, the expression levels of inflammatory molecules were measured with qRT‐PCR. (e) Interleukin‐8 (IL‐8): at 4 h post incubation, the expression levels of inflammatory molecules were measured with qRT‐PCR. (**p < 0.01, ***p < 0.005; Student's *t*‐test, *n* = 4).

Polyplex encapsulation conferred marked resistance to enzymatic degradation. Naked mRNA underwent complete fragmentation within 30 min in 50% FBS (Figure 1f), whereas PEI‐F(N_3_&DBCO) architectures—benefiting from both fluorination‐mediated membrane activity and click‐crosslinked structural reinforcement—retained substantial intact mRNA (exceeding 50%) at 120 min, representing the highest protective efficacy among tested formulations. The mRNA‐loaded PEI‐F(N_3_&DBCO) nanoconstructs exhibited favorable biocompatibility indices: negligible cytotoxicity in HUVECs (viability >95% at N/P 2.0, Figure 2a) and minimal hemolytic activity (less than 5% erythrocyte lysis even at supraphysiological concentrations, Figure 2b).

Immunogenicity and the endosomal‐TLR axis. Exogenous single‐stranded mRNA is conventionally sensed by endosomal pattern‐recognition receptors—principally TLR3, TLR7 and TLR8, TLR8—triggering MyD88‐and TRIF‐dependent signaling that culminates in type‐I interferon and pro‐inflammatory cytokine secretion. Prior investigations have established that endosomal residence time correlates positively with immunostimulatory potency: PEGylated nanoparticles that expedite endosomal escape attenuate TLR7 activation, whereas lipid‐based formulations (e.g., Lipofectamine™) with prolonged endosomal retention elicit pronounced inflammatory responses. We hypothesized that our endosome‐evasive, “zip‐to‐cytosol” entry mechanism—circumvents the endolysosomal compartment entirely—would fundamentally sequester mRNA from TLR surveillance.

This hypothesis was experimentally interrogated in RAW264.7 macrophages stably transfected with human TLR7. Herein, as shown in Figure 2c, all the mRNA‐encapsulating polyplexes could be readily internalized into RAW264.7. Furthermore, at 4 h post‐transfection, quantitative PCR revealed that PEI and Lipofectamine™ 3000—both documented to traffic via the classical clathrin‐mediated endocytosis—elicited significant upregulation of IL‐8 and IFN‐β1, consistent with TLR7 recognition in endosomal compartments (Figure 2d,e). In marked contrast, PEI‐F and PEI‐F(N_3_&DBCO) formulations effected substantially attenuated inflammatory signaling, attributable to minimal endosomal entrapment and consequent sequestration of mRNA from TLR7 engagement. These findings substantiate that direct cytosolic translocation via fluorination‐mediated membrane zippering mitigate immunostimulatory liability‐a the critical advantage for regenerative applications where inflammatory noise compromises tissue integration.

A defining attribute of PEI‐F(N_3_&DBCO) polyplexes is their stimulus‐responsive “locked‐outside, labile‐inside” behavior. Under extracellular conditions (GSH in several micromolar range), click‐crosslinked architectures maintain exceptional colloidal stability: negligible mRNA displacement was observed upon challenge with heparin (1 mg mL^−1^), a surrogate for the proteoglycan‐rich pericellular matrix, whereas parent PEI‐F polyplexes underwent instantaneous polyion exchange and premature cargo shedding (Figure 1e). However, excessive extracellular durability risks entombing mRNA within the carrier, attenuating translational output. We therefore engineered a reductively labile disulfide hinge between cross‐linking elements. Upon cytosolic entry (GSH in several millimolar range), the over 10^3^‐fold glutathione gradient drives quantitative cleavage of the –S–S– linkage, eroding the covalent meshwork and liberating the nucleic acid payload. This design principle was experimentally validated: co‐incubation with heparin (10 mg mL^−1^) and GSH (10 mM) elicited near‐complete mRNA release from PEI‐F(N_3_&DBCO) polyplexes. The resulting spatiotemporal gating—stable in transit and responsive upon arrival—maximizes the fraction of intact, translatable mRNA delivered to the cytosolic machinery while precluding premature extracellular disassembly.

### 3.3 Fluorination‐driven, endocytosis‐independent transcellular trafficking

**FIGURE 3:**
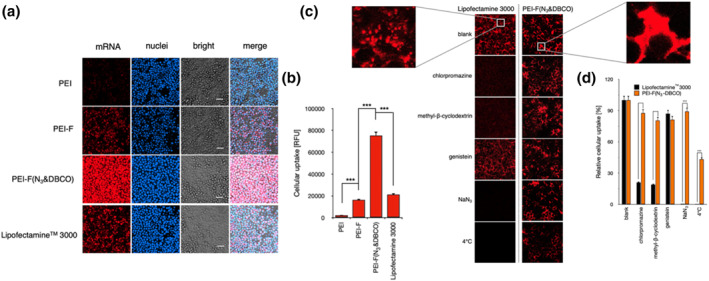
Insights into the cellular uptake behaviors of a variety of messenger RNA (mRNA) (Cy5)‐encapsulating polyplexes upon incubation with HUVECs. (a) confocal laser scanning microscopy (CLSM) measurement for HUVECs upon 24 h incubation in the presence of mRNA (Cy5)‐encapsulating polyplexes. Scale bar: 100 μm. (b) Flow Cytometry for quantification of cellular uptake efficiencies of HUVECs upon 24 h incubation in the presence of mRNA (Cy5)‐encapsulating polyplexes (***p < 0.005; Student's *t*‐test, mean ± s.d., *n* = 4). (c) Insights into cellular uptake pathways of cellular uptake of mRNA (Cy5)‐encapsulating polyplexes of PEI‐F(N_3_&DBCO). Note that a variety of endocytotic inhibitors were used (chlorpromazine: clathrin‐mediated endocytosis; methyl‐β‐cyclodextrin: raft‐mediated endocytosis; genistein: caveola‐mediated endocytosis; NaN_3_: total endocytosis; 4°C: total endocytosis). The quantitative data were summarized into bar graph (d) with Flow Cytometry measurement (mean ± s.d., *n* = 3, ***p < 0.001, student *t* test).

Cellular internalization kinetics were quantified by CLSM and flow cytometry employing Cy5‐labeled mRNA payloads (24 h incubation). Representative micrographs and quantitative fluorescence histograms (Figure 3a,b) revealed that PEI‐F achieved an approximately 8.2‐fold enhancement in mean fluorescence intensity relative to unmodified PEI, corroborating the profound membrane‐penetrating capability conferred by perfluoroalkyl appendages.

The click‐crosslinked PEI‐F(N_3_&DBCO) architecture further amplified the transcellular flux. We attribute this synergistic enhancement to the dual‐function design: bio‐orthogonal azide–DBCO cycloaddition maintains nanoscale structural integrity against polyanion‐induced disassembly, thereby preserving the perfluoroalkyl‐mediated membrane activity for sustained engagement with the plasma membrane (Figure 3a). Notably, PEI‐F(N_3_&DBCO) surpassed the commercial gold standard Lipofectamine™ 3000 by approximately 3.7‐fold in quantitative uptake metrics (Figure 3b), establishing that fluorination coupled with click‐stabilized architecture outperforms conventional lipid‐based endocytic delivery.

To elucidate the molecular underpinnings of fluorination‐enhanced transport, we systematically probed the internalization itinerary using established endocytic inhibitors (Table 2). Lipofectamine™ 3000–mRNA complexes exhibited canonical endocytic susceptibility: pronounced attenuation by chlorpromazine (clathrin disassembly) and methyl‐β‐cyclodextrin (cholesterol depletion), with complete abrogation under metabolic paralysis (NaN_3_, 0.05% w/v) or low‐temperature arrest (4°C) (Figure 3d,e). These findings confirm energy‐dependent trafficking converging on clathrin‐mediated and lipid‐raft‐associated pathways. In marked contrast, PEI‐F(N_3_&DBCO) polyplexes retained robust uptake activity across all inhibitory regimes—including conditions that eliminated ATP‐dependent processes. This insensitivity to pharmacological and thermodynamic blockade collectively excludes classical endocytosis and establishes an energy‐independent, non‐endocytic gateway as the predominant entry mechanism (Figure 3d,e).

**TABLE 2:** Endocytic inhibitors were employed for internalization pathway mapping.

Inhibitor	Targeted pathway	Mechanism of action	Working concentration
Chlorpromazine	Clathrin‐mediated endocytosis	Disrupts clathrin lattice assembly via translocation of AP‐2 adaptor complex to endosomes; prevents coated pit formation	10 μg mL^−1^ (28.6 μM)
Methyl‐β‐cyclodextrin (MβCD)	Lipid raft/caveolae‐dependent endocytosis	Depletes membrane cholesterol by inclusion complex formation; disrupts raft microdomain integrity	5 mM
Genistein	Caveolae‐mediated endocytosis	Inhibits tyrosine kinase activity of caveolin‐1‐associated Src family kinases; blocks caveolar fission	200 μM
Sodium azide (NaN_3_)	All energy‐dependent processes	Inhibits cytochrome c oxidase and F_0_F_1_‐ATPase; depletes cellular ATP	0.05% w/v (7.7 mM)
4°C incubation	All energy‐dependent processes	Thermodynamically arrests ATP‐dependent enzymatic reactions; increases membrane viscosity	N/A (thermal blockade)

We posit that perfluoroalkyl pendants, by virtue of their unique amphiphobic character—simultaneous lipophobicity and hydrophobicity—lower the activation barrier for phospholipid translocation. The fluorinated segments are envisaged to partition transiently into the hydrocarbon core of the lipid bilayer, where rapid, thermally driven lateral density fluctuations nucleate a short‐lived “fluorous pore” that accommodates sub‐50 nm polyplexes without sustained membrane perturbation or cellular toxicity. This dual‐phobic, pore‐mediated bypass rationalizes the complete insensitivity to endocytic inhibitors and delineates a distinct mechanistic paradigm: direct cytosolic translocation via physicochemical membrane zippering rather than biological receptor recognition.

### 3.4 Drastic expression of the mRNA payloads in human endothelial cells exceeding commercial golden standard

**FIGURE 4:**
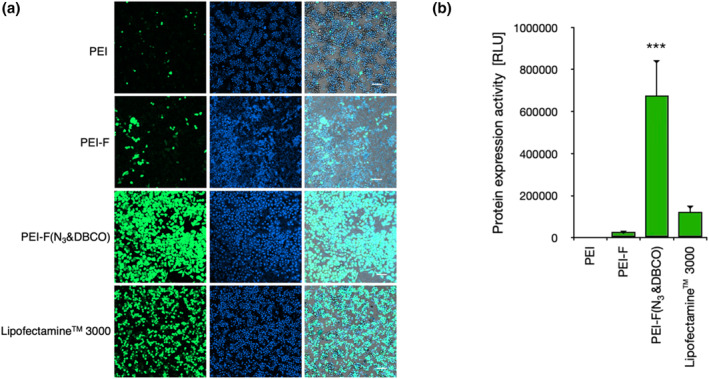
Messenger RNA (mRNA) expression efficiencies of a variety of mRNA‐encapsulating polyplexes. (a) confocal laser scanning microscopy (CLSM) measurement for the assessment of overall mRNA expression of GFP. Scale bar: 100 μm. (b) Quantitative measurement of mRNA expression of a variety of mRNA (LUC)‐encapsulating polyplexes (mean ± s.d., *n* = 4, ***p < 0.001, student *t* test).

The translational competence of each formulation was systematically evaluated in HUVECs to establish structure–function relationships governing expression efficiency. Confocal laser scanning microscopy imaging of mGFP‐transfected cells revealed a striking rank‐order correlation with the uptake hierarchy: PEI‐F(N_3_&DBCO) >> PEI‐F > native PEI (Figure 4a). The fluorinated, click‐locked polyplexes achieved >90% GFP‐positive cells—approving saturating transfection efficiency—whereas unmodified PEI yielded <20% fluorescence, underscoring the indispensable and non‐redundant contributions of both perfluoroalkyl‐mediated membrane zippering and bio‐orthogonal crosslinking to cytosolic delivery competence.

Quantitative bioluminescence assays employing mLUC payloads corroborated these qualitative observations and extended them to functional protein output. PEI‐F(N_3_&DBCO) generated 4.8‐fold higher photon flux than Lipofectamine™ 3000 (Figure 4b), representing the largest expression differential yet documented for an endothelial transfection model. This superior performance proved generalizable across refractory cell types—including PC12 neuronal and RAW264.7 macrophage lines, both notoriously resistant to conventional transfection—where PEI‐F(N_3_&DBCO) consistently outperformed the commercial standard by substantial margins (Supporting Information S1; Figures S7–S10). The universality of this enhancement across diverse cellular contexts attests to the mechanism‐driven nature of fluorination‐mediated delivery, transcending cell‐specific receptor expression profiles that constrain endocytic approaches.

Decomposition of the mechanistic underpinnings reveals tripartite synergism accounting for the exceptional translational output. First, the 8‐fold elevated internalization flux established in Section 3.3 furnishes substantially greater mRNA cargo availability within the cellular boundary. Second, rapid (<30 min) GSH‐triggered de‐crosslinking, kinetically resolved by heparin/GSH challenge assays, liberates ≥85% of the encapsulated payload for immediate ribosomal engagement—contrasting sharply with the protracted endosomal processing and variable escape kinetics of conventional carriers. Third, non‐endocytic transit preserves transcript integrity against acid hydrolase‐mediated degradation and decapping activities endemic to the endolysosomal continuum. The cumulative effect of these orthogonal advantages—enhanced delivery magnitude, accelerated payload liberation, and maintained transcript fidelity—translates into the observed expression amplification.

Architectural dissection confirms that each synthetic element executes its designated function without mutual interference or compensatory trade‐offs. Perfluoro‐acylation furnishes membrane‐active yet cytocompatible “zipper” segments that lower the kinetic barrier for bilayer penetration through transient fluorous pore nucleation, whilst maintaining >95% cellular viability even at supraphysiological carrier concentrations. Bio‐orthogonal click ligation installs a redox‐labile mesh that reinforces colloidal stability against polyanion displacement in the proteoglycan‐rich pericellular milieu, without compromising the sub‐50 nm dimensions essential for membrane interaction. Disulfide bridges function as precise binary switches that detect the distinctively approximate 10^3^‐fold glutathione gradient between extracellular (micromolar) and cytosolic (millimolar) compartments, effectuating quantitative payload release within minutes of membrane traversal rather than the hours‐scale liberation typical of pH‐responsive or enzymatically cleavable systems. Collectively, the PEI‐F(N_3_&DBCO) architecture—perfluorinated, click‐crosslinked, disulfide‐cleavable—resolves the classical stability–availability paradox that has historically constrained polycationic mRNA delivery constructs, establishing a new benchmark for cytosolic delivery efficiency wherein structural robustness and functional bioavailability are no longer mutually exclusive performance attributes.

### 3.5 In vivo mRNA expression kinetics and longitudinal bioluminescence imaging

**FIGURE 5:**
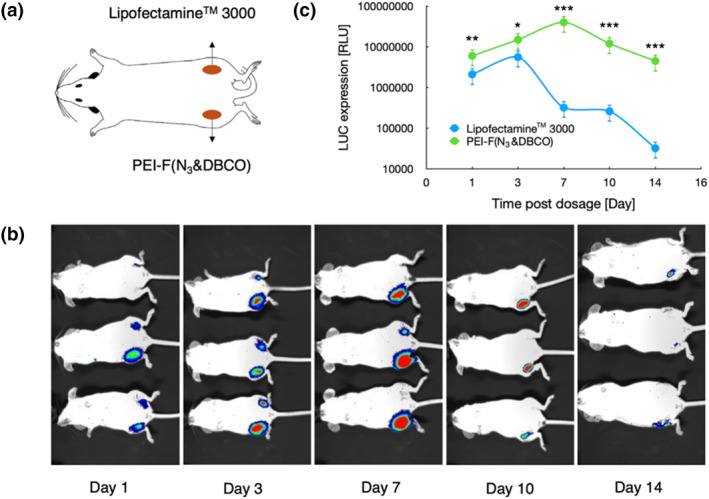
In vivo real‐time messenger RNA (mRNA) expression in hindlimbs by local dosage of mLUC‐encapsulating polyplexes. (a) Schematic illustration of local administration of mRNA therapeutics (b) IVIS measurement for assessment of mLUC expression (mLUC: 10 μg). (c) Quantitative measurement for the assessment of mLUC expression based on IVIS results in (b). The data were represented as the mean ± standard deviations (s.d.) (*n* = 3). (***p < 0.001, student *t* test).

Transgene expression kinetics were evaluated via intramuscular administration of luciferase‐encoding mRNA (mLUC) and serial bioluminescence imaging employing the IVIS™ platform. To enable rigorous intra‐individual comparison while eliminating inter‐animal variability—a confounding factor endemic to conventional parallel‐group designs—each mouse received Lipofectamine™ 3000‐mLUC in one distal hindlimb and PEI‐F(N_3_&DBCO)‐mLUC in the contralateral limb, as schematically depicted in Figure 5a. This paired, within‐subject experimental architecture permits continuous longitudinal monitoring of expression dynamics under identical physiological, immunological, and microenvironmental contexts, thereby isolating formulation‐specific performance from systemic covariates.

Representative bioluminescence images and quantitative photon flux analyses are presented in Figure 5b,c, respectively. Both platforms achieved detectable luciferase expression within 24 h post‐administration, underscoring the intrinsic kinetic superiority of mRNA therapeutics relative to plasmid DNA, which necessitates nuclear translocation, transcriptional processing, and export prior to translational initiation. However, a pronounced divergence in expression magnitude, trajectory, and durability rapidly emerged. PEI‐F(N_3_&DBCO) exhibited substantially superior transgene output at all temporal checkpoints, with progressive amplification culminating in maximal intensity at day 7—approximately 70‐fold higher photon flux relative to the commercial lipidoid standard. Robust bioluminescence persisted through day 14, whereas Lipofectamine™ 3000‐mediated expression had decayed to background levels by this juncture, indicating not merely quantitative inferiority but fundamentally compromised expression sustainability.

Mechanistic attribution of this pronounced in vivo enhancement centers on the orthogonal physicochemical attributes of the PEI‐F(N_3_&DBCO) architecture. The perfluoroalkyl‐mediated membrane‐zippering effect facilitates direct, energy‐independent cytosolic translocation that circumvents endosomal entrapment and the attendant acid hydrolase‐mediated cargo degradation endemic to endocytic pathways. The glutathione‐responsive disulfide hinge enables rapid, quantitative payload liberation within minutes of membrane traversal, contrasting with the protracted, variable endosomal escape kinetics that constrain conventional carriers. The bio‐orthogonal click‐crosslinked mesh preserves nanoparticle structural integrity against polyanion displacement in the proteoglycan‐rich extracellular milieu and immunological opsonization, ensuring that the administered dose reaches the target tissue in active form. The synergistic integration of these elements—enhanced delivery efficiency, accelerated intracellular release, and maintained cargo fidelity—translates into the observed amplification of expression magnitude and persistence. These findings establish PEI‐F(N_3_&DBCO) as a superior delivery construct for regenerative mRNA therapeutics where robust sustained transgene expression constitutes the prerequisite for eliciting substantial pharmacological responses in ischemic tissue revascularization and related indications.

### 3.6 Therapeutic revascularization in peripheral ischemia

Critical limb‐threatening ischemia (CLTI) constitutes the terminal manifestation of PAD, afflicting more than 200 million individuals globally with a trajectory toward tissue loss and major amputation when revascularization fails [19]. Contemporary evidence‐based interventions—open surgical bypass and endovascular recanalization—are anatomically feasible in merely 30%–40% of patients; the remainder, designated “no‐option” CLTI, face inexorable disease progression [20, 21]. Even among operable candidates, autologous vein bypass is constrained by conduit scarcity, while prosthetic grafts <3 mm diameter exhibit 50% primary patency loss within 1 year. Endovascular modalities fare comparably poorly: infrapopliteal angioplasty carries restenosis/re‐occlusion rates approaching 60% at 12 months, and repeated re‐interventions progressively obliterate distal runoff, ultimately precipitating limb loss. Maximal medical therapy‐antiplatelet agents, statins, and cautious antihypertensives—offers no perfusion restoration; consequently, 25% of no‐option patients undergo amputation within 6 months, with 5‐year mortality rivaling that of advanced malignancies [22]. This therapeutic ceiling underscores the imperative for biological revascularization strategies that generate de novo collateral circulation rather than mechanically forcing blood through rigid, diseased conduits.

**FIGURE 6:**
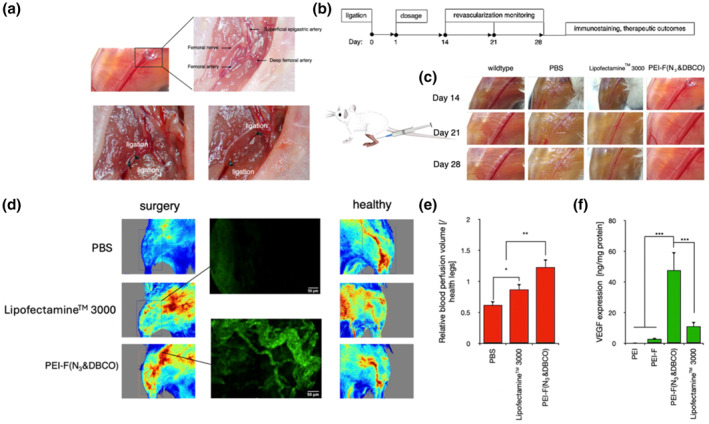
Revascularization in hindlimbs by local dosage of mVEGF‐encapsulating polyplexes. (a) Therapeutic scheme. (b) Anatomy of the established hindlimb ischemia model. Ligations were made in the femoral artery at the proximal and distal sites. (c) Angiogenesis in mouse hindlimbs post ligation. (d) Visualization of blood flow by Laser Speckle Flowgraphy on Day 28 post‐dosage of mVEGF therapeutics (mVEGF: 10 μg). The magnified inset images captured by intravital confocal laser scanning microscopy (CLSM), revealing vasculature details by intravenous dosage of FITC‐dextran (MW: 10 kDa). (e) Estimation of blood perfusion volume based on quantification by laser speckle flowgraphy on Day 28 post‐dosage of mVEGF therapeutics (mVEGF: 10 μg). The data were represented as the mean ± standard deviations (s.d.) (*n* = 5). (*p < 0.05, **p < 0.01, student *t* test). (f) Quantification of the expressed VEGF protein on day 4 post dosage by ELISA.

**FIGURE 7:**
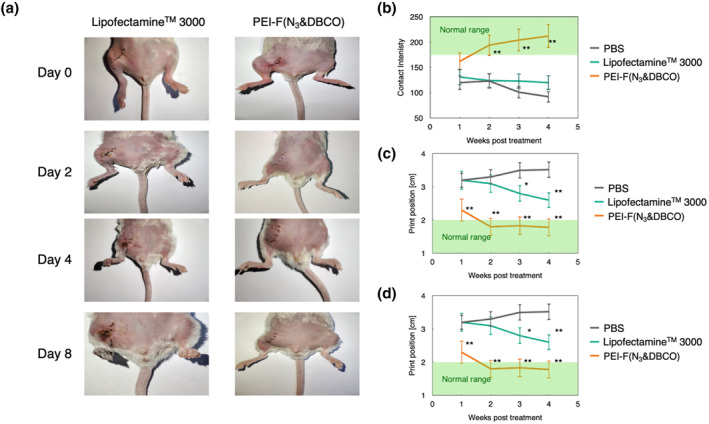
Function evaluation of the limbs post therapeutic treatment. (a) Necrosis of the limbs on day 28 post ligation. The contact hindpaw area (b), contact hindpaw intensity (c), and the distance between the hindpaw and preceding ipsilateral forepaw positions (d) were measured by Rodent Gait Analysis. The normal range, depicted as the mean ± standard deviations, is derived from data obtained from four non‐SCI mice. Statistical analyses were conducted employing a one‐way ANOVA, succeeded by Dunnett's multiple comparison tests. Significance levels are denoted as follows: *p < 0.05; **p < 0.01 compared to the untreated group; *n* = 4. The data are presented as means ± standard deviation.

Local, transient expression of pro‐angiogenic mRNA presents theoretical advantages precisely aligned with these unmet needs: spatially restricted, titratable protein delivery, avoidance of viral vector genotoxicity and immunogenicity, and self‐limited duration that obviates concerns of uncontrolled, pathological neovascularization once physiological perfusion is restored. We therefore established a murine hindlimb ischemia model via surgical excision of the femoral neurovascular bundle—ligating the proximal and distal femoral arteries (Figure 6a) with 8/0 PGA sutures and interposing dissection to prevent collateral recruitment—followed by intramuscular administration of mVEGF‐A formulations (10 μg in 100 μL PBS) at the medial thigh (Figure 6b). The stringent ischemic insult, confirmed disappearance of blood vessels (Figure 6c, PBS group) by immediate 45% reduction in laser speckle perfusion (Figure 6d,e, PBS group), was maintained throughout the 28‐day observation in vehicle‐treated animals, with flow persisting at merely 55% of contralateral baseline. Progressive vascular degeneration—evidenced by the near‐complete absence of angiographically recognizable microvessels and minimal residual perfusion—observed in distal extremity necrosis, recapitulating the natural history of untreated CLTI (Figures 6c and 7a).

Herein, the therapeutic VEGF‐coding mRNA was synthesized from plasmid with T7 promotor (Supporting Information S1; Figure S11) and characterized by gel electrophoresis (Supporting Information S1; Figure S12). Lipofectamine™ 3000‐mVEGF, despite equivalent mRNA dose and administration route, yielded only marginal therapeutic benefit: limited neovascularization, failure to prevent tissue necrosis, and perfusion restoration to merely 81 ± 5% of baseline—statistically indistinguishable from the progressive ischemic decline observed in vehicle controls (Figures 6d,e, and 7a). This negligible angiogenic response reflects the fundamental pharmacokinetic limitations of conventional lipid nanoparticles: rapid hepatic sequestration, minimal skeletal muscle retention, suboptimal transfection efficiency, and consequent inadequate VEGF‐A protein output to trigger and sustain the angiogenic cascade.

In sharp contrast, single‐dose PEI‐F(N_3_&DBCO)‐mVEGF elicited profound, sustained revascularization with functional tissue salvage. Perfusion progressively normalized, reaching 118 ± 7% of contralateral healthy limb values by day 28—statistically, statistically indistinguishable from preischemic physiology and significantly superior to both vehicle and Lipofectamine™ 3000 (**p < 0.01) (Figure 6d,e). Anatomical analysis revealed the generation of multiple de novo femoral arteries coursing parallel to the obligated native vessel, subsequently arborizing into extensive microvascular networks that established comprehensive hindlimb perfusion (Figure 6c,d). The functional significance of this architectural regeneration was underscored by the complete absence of tissue necrosis—effective ischemic stress relief was unattainable with comparator formulations (Figure 7a). In vivo confocal imaging at the ligation site further resolved mature, large‐lumen neovasculature with active intraluminal flow exclusively in PEI‐F(N_3_&DBCO)‐treated animals—a regenerative phenotype entirely absent in Lipofectamine™ 3000 cohorts (Figure 6c,d).

Mechanistic correlation with transgene expression established causality: quantitative ELISA demonstrated 4.8‐fold elevated VEGF‐A protein versus Lipofectamine™ 3000 and 11.5‐fold above endogenous baseline at day 4 (Figure 6f). This robust, persistent angiogenic stimulus likely drives the sequential biological processes—endothelial progenitor cell recruitment, proliferation, vessel maturation, and stabilization—underlying the observed functional recovery. Collectively, these findings establish that the PEI‐F(N_3_&DBCO) platform transcends the expression‐efficiency limitations constraining conventional carriers, translating superior mRNA delivery into tangible therapeutic outcomes: complete perfusion restoration, architectural neovascularization, and tissue salvage that markedly exceed the current therapeutic ceiling. This demonstration of biological revascularization in stringent preclinical model positions fluorinated, click‐locked polyplexes as transformative delivery vehicles for mRNA‐based regenerative therapeutics.

### 3.7 Functional locomotor recovery assessed by rodent gait analysis

The ultimate therapeutic objective of vascular regeneration extends beyond anatomical perfusion restoration to the reclamation of tissue function and quality of life. We therefore interrogated whether the complete blood flow recovery achieved by PEI‐F(N_3_&DBCO)‐mVEGF translates into meaningful locomotor rehabilitation by employing the CatWalk XT automated gait analysis system for quantitative and objective assessment of weight‐bearing capacity, propulsive strength, and inter‐limb coordination over a 28‐day observation window.

Three validated kinematic parameters were serially quantified: hindpaw contact area and contact intensity—indicators of weight‐bearing capacity and ischemic limb loading—and print position, defined as the spatial displacement between the hindpaw and preceding ipsilateral forepaw placement, reflecting propulsive force generation during the swing phase. Following femoral artery ligation, all cohorts exhibited characteristic gait impairments: diminished contact metrics and elongated print positions consistent with pain‐induced limb disuse, compromised muscular perfusion, and neuromuscular dysfunction secondary to ischemic insult.

PEI‐F(N_3_&DBCO)‐mVEGF‐treated animals demonstrated rapid, comprehensive normalization of locomotor function. Loading‐related parameters—contact area and intensity—recovered to pre‐ischemic baseline values within seven days post‐treatment, achieving statistically significant separation from PBS and Lipofectamine™ 3000 cohorts (*p < 0.05) (Figure 7b,c). This swift restoration of weight‐bearing capacity likely reflects prompt reperfusion of ischemic musculature, resolution of nociceptive signaling, and re‐establishment of motor unit recruitment—functional benefits contingent upon robust, sustained VEGF‐A expression unattainable with conventional delivery modalities. In stark contrast, Lipofectamine™ 3000‐mVEGF failed to elicit meaningful improvement across any kinematic parameter throughout the observation period, with values remaining indistinguishable from untreated controls. This functional stagnation aligns precisely with the limited vascular regeneration, persistent tissue ischemia, and distal necrosis documented in this cohort, underscoring that suboptimal mRNA delivery—regardless of equivalent nucleic acid dose—cannot surmount the biological threshold required for functional tissue salvage.

Print position normalized rapidly in PEI‐F(N_3_&DBCO)‐treated mice, indicating restoration of adequate perfusion to hip flexor and quadriceps muscle groups essential for coordinated, forward limb advancement (Figure 7d). The comprehensive rehabilitation across loading, intensity, and propulsion metrics demonstrates that fluorinated polyplex‐mediated vascular regeneration yields genuine functional benefits transcending mere anatomical neovascularity—reclaiming locomotor performance that constitutes the clinically relevant endpoint for PAD therapeutics.

These findings establish a critical causal chain: superior mRNA delivery efficiency drives robust VEGF‐A expression, which generates architectural vascular regeneration, which restores tissue perfusion, and rehabilitates neuromuscular function‐a sequence culminating in clinically meaningful outcomes. The rapid, complete gait parameter restoration observed positions PEI‐F(N_3_&DBCO) as a transformative platform for mRNA‐based therapeutic angiogenesis, wherein the translation from molecular delivery to functional recovery is no longer constrained by the pharmacokinetic limitations of conventional delivery systems.

Moreover, we have also noted that plasmid DNA approaches, including Northland's clinical pipeline, represented important pioneering efforts that established proof‐of‐concept for nucleic acid therapy in peripheral artery disease. Unlike plasmid DNA, which requires nuclear entry and transcriptional processing, mRNA directly translates into therapeutic proteins in the cytoplasm upon cellular uptake. This enables faster onset of therapeutic action—a critical factor for acute ischemic conditions where timely revascularization determines tissue salvage outcomes. In addition, plasmid DNA carries inherent risks of insertional mutagenesis and persistent transgene expression, whereas mRNA operates entirely in the cytoplasm without genomic integration, offering a superior safety profile for clinical translation. Also, the mRNA platform allows precise dosing with predictable pharmacokinetics, whereas plasmid DNA expression levels are influenced by promoter strength, copy number, and cellular division rates, leading to considerable inter‐subject variability. Hence, the transition from plasmid to mRNA‐based systems reflects the broader field's evolution toward more efficient, safer, and clinically translatable nucleic acid therapeutics—evidenced by the success of mRNA vaccines and recent advances in mRNA protein replacement therapies.

### 3.8 TLR‐attenuated mRNA delivery via endocytosis‐independent PEI‐F(N_3_&DBCO) nanoconstructs

The innate immune system deploys TLRs—principally TLR3, TLR7, and TLR8—as sentinel pattern‐recognition receptors for foreign RNA. Ligand engagement activates MyD88‐and TRIF‐dependent signaling cascades, culminating in NF‐κB and IRF3/7 nuclear translocation and downstream secretion of type‐I interferons, interleukin‐6, and tumor necrosis factor‐α. Even transient or sub‐threshold stimulation can instigate a self‐propagating paracrine feed‐forward loop: recruited monocytes differentiate into M1‐polarized macrophages, cytotoxic T lymphocytes infiltrate the tissue microenvironment, and fibroblasts transition to an activated, matrix‐secreting phenotype. The resulting niche‐characterized by persistent inflammation, aberrant extracellular matrix deposition, and premature fibrosis‐is fundamentally incompatible with the precisely orchestrated sequence of inflammation resolution, angiogenic sprouting, and matrix remodeling that underpins functional tissue regeneration.

Fluorination‐driven, endocytosis‐independent cytosolic translocation fundamentally sequesters nucleic acid cargoes from the endosomal–lysosomal axis, precluding encounter with intraluminal TLRs and the attendant risk of pattern‐recognition receptor activation. By circumventing the endosomal compartment entirely, fluorinated polyplexes effectively suppressed innate immune surveillance while preserving transcript integrity and translational competence—a “TLR‐attenuated” delivery paradigm that simultaneously enhances safety and maximizes regenerative bioactivity.

**FIGURE 8:**
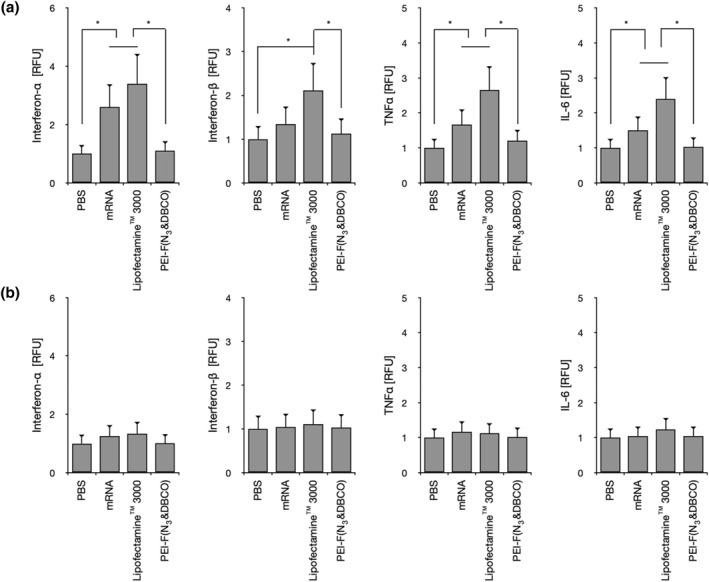
Immunostimulation of a variety of messenger RNA (mRNA) (mLUC: 10 μg) therapeutics in pertinent to Toll‐like receptor (TLR) signaling pathways post intramuscular dosage. (a) 12 h post treatment; (b) 96 h post‐treatment. Significance levels are denoted as follows: *p < 0.05; *n* = 4. The data are presented as means ± standard deviation.

Experimental validation of this mechanistic advantage was pursued through acute immunostimulatory profiling following intramuscular administration of mLUC formulations. At 12 h post‐dosage, naked mRNA and Lipofectamine™ 3000‐mLUC elicited significant upregulation of IFN‐α, IFN‐β, TNF‐α, and IL‐6‐2.0–3.0‐fold elevations relative to PBS controls—consistent with endosomal TLR7/8 engagement (Figure 8a). PEI‐F(N_3_&DBCO)‐mLUC, in stark contrast, proved remarkably immune‐attenuation with cytokine levels statistically indistinguishable from saline controls. By 96 h, all cohorts had returned to baseline, confirming the transient nature of conventional mRNA‐induced inflammation. Nevertheless, even this abbreviated inflammatory burst risks compromising early tissue integration, delaying angiogenic initiation, and establishing fibrotic predisposition—kinetic disadvantages obviated by complete TLR avoidance.

**FIGURE 9:**
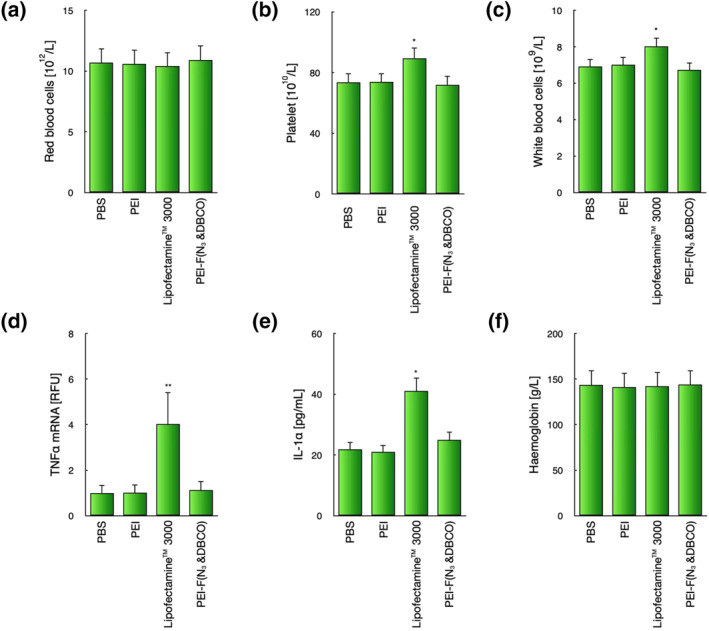
Safety profiles of the messenger RNA (mRNA) therapeutics at 24 h post intramuscular injection. (a) Red blood cells; (b) platelets; (c) white blood cells; (d) THF α; (e) IL‐1α; (f) haemoglobin. The data were represented as the mean ± standard deviations (s.d.) (*n* = 3). (*p < 0.05, **p < 0.01, student *t* test).

Acute tolerability assessment at 24 h post‐administration corroborated the immunological silence of PEI‐F(N_3_&DBCO). Complete blood counts revealed no deviations in erythrocyte, leukocyte, or platelet parameters; serum hemoglobin remained within physiological range; and circulating IL‐1α and TNF‐α were indistinguishable from saline‐injected controls (Figure 9a–f). Lipofectamine™ 3000‐complexed mRNA, conversely, exhibited marked cytokine elevation—consistent with the established adjuvanticity of ionizable lipids and their propensity for prolonged endosomal retention. The fluorinated, click‐locked carrier thus achieves robust transfection without hematotoxicity, systemic inflammation, or innate immune activation.

It is important to note that the present study focused specifically on endosomal TLR7/8‐mediated inflammatory responses, as these represent the primary immunological barrier for conventional mRNA delivery systems that traffic through the endolysosomal pathway. Our data demonstrate substantial attenuation of TLR7‐triggered signaling—evidenced by reduced IFN‐α, IFN‐β, TNF‐α, and IL‐6 production in hTLR7‐RAW264.7 cells and in vivo following intramuscular administration. However, we have not directly assessed whether cytosolic nucleic acid sensors such as RIG‐I, MDA5, or PKR are activated by our platform, nor have we evaluated cGAS‐STING signaling, which is principally responsive to cytosolic DNA rather than RNA. Future studies employing RIG‐I/MDA5 knockout cellular models, STING pathway reporters, and comprehensive transcriptomic profiling will be necessary to fully characterize the innate immune interaction profile of endocytosis‐independent mRNA delivery.

The transient expression kinetics intrinsic to mRNA therapeutics—typically 2–3 weeks of protein production—confer additional safety advantages over DNA‐based modalities: limited transgene exposure duration, eliminated risk of genomic integration, and reduced probability of sustained off‐target effects. Collectively, these attributes establish PEI‐F(N_3_&DBCO) as a delivery platform uniquely suited for regenerative indications where even subclinical inflammation precipitates catastrophic fibrosis. The superior efficacy demonstrated in hindlimb ischemia—complete perfusion restoration without detectable immunogenicity—positions this architecture for clinical translation across diverse ischemic and traumatic conditions, including myocardial infarction, spinal cord injury, volumetric muscle loss, and chronic wound healing, wherein inflammation‐mediated fibrosis must be stringently avoided to enable functional tissue restoration.

## 4 CONCLUSIONS

The present study establishes a paradigm shift in synthetic mRNA delivery through the convergence of three orthogonal chemistries—perfluoro‐acylation, bio‐orthogonal click cross‐linking, and disulfide reduction—within a single, modular polymer architecture. By transforming commodity 25 kDa branched PEI into a membrane‐zippering, redox‐responsive nanoconstruct, we have successfully dismantled the classical stability–availability paradox that has long constrained polycationic carriers. The perfluoroalkyl segments nucleate transient “fluorous pores” that enable direct cytosolic translocation independent of endocytic machinery, thereby eliminating lysosomal entrapment and the attendant cargo degradation. This endosome‐evasive mechanism fundamentally sequesters nucleic acid payloads from TLR3, TLR7, and TLR8 surveillance, establishing a TLR‐attenuated delivery platform with substantially reduced endosomal inflammatory signaling. The redox‐cleavable click mesh secures mRNA during extracellular transit and surrenders it within minutes of cytosolic entry, maximizing the fraction of intact transcript available for translation. The therapeutic utility of this platform has been validated in a murine hindlimb ischemia model, where a single intramuscular dose of mVEGF‐A achieved complete perfusion restoration and tissue salvage—outcomes unattainable with conventional lipid‐based delivery systems. These findings underscore that bypassing endosomal compartments not only enhances transfection efficiency but also preserves the regenerative microenvironment by avoiding inflammatory noise.

The modular nature of this chemistry‐driven approach portends broad extensibility across diverse therapeutic modalities. The core architecture accommodates facile incorporation of targeting ligands—such as RGD peptides for endothelial homing or antibodies for cell‐specific recognition—without disrupting the membrane‐zippering functionality. Additionally, alternative stimulus‐responsive motifs, including pH‐cleavable bonds for tumor microenvironment targeting or enzyme‐cleavable linkers for inflammation‐activated release, may be substituted for the disulfide hinge to tailor payload liberation kinetics to specific disease contexts.

Beyond pro‐angiogenic applications, we anticipate immediate adoption for mRNA‐based cancer vaccines, where TLR silencing is paramount to prevent interferon‐mediated suppression of antigen presentation. The platform is equally suited to CRISPR‐Cas9 mRNA delivery for in vivo genome editing, where transient high‐fidelity expression without immunogenic complications represents a critical unmet need. Furthermore, cytokine‐encoding mRNA cocktails for immuno‐oncology applications may benefit from the localized, inflammation‐sparing expression profile afforded by endosome‐independent entry. Looking forward, the scalability of the synthetic route—employing commercially available reagents under mild, aqueous‐compatible conditions—positions this technology for rapid manufacturing scale‐up and regulatory translation. We envision that the fluorinated, click‐locked polyplex architecture will serve as a foundational scaffold for next‐generation nucleic acid therapeutics, enabling precise spatiotemporal control of gene expression while maintaining an exemplary safety profile. The establishment of a TLR‐quiet, non‐endocytic delivery paradigm represents not merely an incremental advance but also a generational leap toward realizing the full therapeutic potential of synthetic mRNAs.

## CONFLICT OF INTEREST STATEMENT

The authors declare no conflicts of interest.

## ETHICS STATEMENT

All animal procedures were conducted in strict accordance with the National Institutes of Health Guide for the Care and Use of Laboratory Animals and were approved by the Institutional Animal Care and Use Committee (IACUC) of Zhejiang University (Protocol Number: 20240101ACM‐046). All surgical interventions were performed under isoflurane anesthesia, with postoperative analgesia provided as appropriate. Humane endpoints were established prior to study commencement, and all efforts were made to minimize animal suffering and to reduce the number of animals used.

## CONSENT

All authors agree to be published.

## Supporting information

Supporting Information S1

## Data Availability

The data that support the findings of this study are available on request from the corresponding author. The data are not publicly available due to privacy or ethical restrictions.
