# Supplementary material for: Endocytosis‐independent cytosolic entry of messenger RNA via fluorous bilayer zippering attenuating Toll‐like receptor signaling and enables ischemic tissue salvage
Source: Smart Mol. 2026 Jul 21:e70085. Online ahead of print. doi: 10.1002/smo2.70085 (PMC13398659; doi:10.1002/smo2.70085)
Supplement: Supplementary file 1 — Supporting Information S1 [file SMO2-9999-0-s001.docx]

***Supporting Information***

**Endocytosis-Independent Cytosolic Entry of mRNA via Fluorous Bilayer Zippering Eliminates TLR Signaling and Enables Ischemic Tissue Salvage**

Yue Wang^a,b,c,d,#^, Haitao Xie^e,#^, Guoqing Xiang^f,#^, Yanhua Li^g^, Changgui Tong^g^, Hongyan Cui^h^, Qixian Chen^a,b,h,*^, Haidong Li^b,i,*^, and Yan Zhao^a,b,d,*^

^a^Department of Gastric Surgery, Cancer Hospital of Dalian University of Technology (Liaoning Cancer Hospital & Institute), No. 44 Xiaoheyan Road, Dadong District, Shenyang 110042, China

^b^Provincial Key Laboratory of Interdisciplinary Medical Engineering for Gastrointestinal Carcinoma, Cancer Hospital of Dalian University of Technology (Liaoning Cancer Hospital & Institute), No. 44 Xiaoheyan Road, Dadong District, Shenyang 110042, China

^c^Immunotherapy & Tumor Metabolism-Microecology Research Unit, Cancer Hospital of Dalian University of Technology (Liaoning Cancer Hospital & Institute), No. 44 Xiaoheyan Road, Dadong District, Shenyang 110042, China

^d^Department of Gastric Surgery, Cancer Hospital of China Medical University, No. 44 Xiaoheyan Road, Dadong District, Shenyang 110042, China

^e^Department of International Medical Services, Cancer Hospital of Dalian University of Technology (Liaoning Cancer Hospital & Institute), No.44 Xiaoheyan Road, Dadong District, Shenyang 110042, China

^f^Department of Endoscopy, Cancer Hospital of Dalian University of Technology (Liaoning Cancer Hospital & Institute), No.44 Xiaoheyan Road, Dadong District, Shenyang 110042, China

^g^The Second Aﬃliated Hospital of Dalian Medical University, Dalian 116023, China

^h^Innovation Center of Yangtze River Delta, Zhejiang University, Jiaxing 314100, China

^i^MOE Key Laboratory of Bio-Intelligent Manufacturing, School of Bioengineering, Dalian University of Technology, Dalian 116024, China.

^#^These authors contributed equally in this work.

*All correspondence should be addressed to Qixian Chen (E-mail: plasmid@zju.edu.cn); Haidong Li (Email: lihd@dlut.edu.cn); Yan Zhao (zhaoyan@cancerhosp-ln-cmu.com)

**
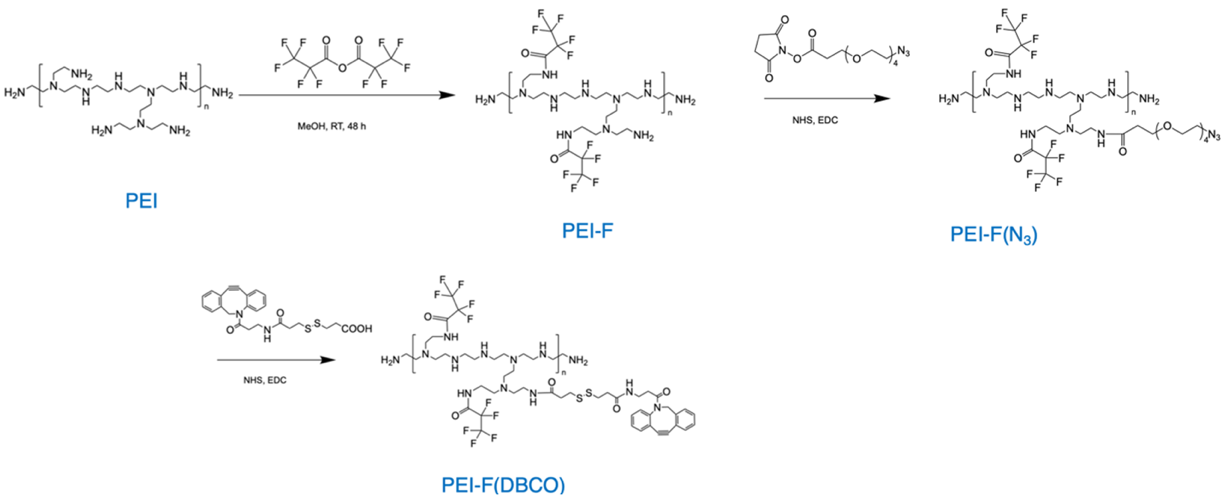
**

**Figure S1** Synthetic route of click-chemistry partners of fluorinated PEI.

*
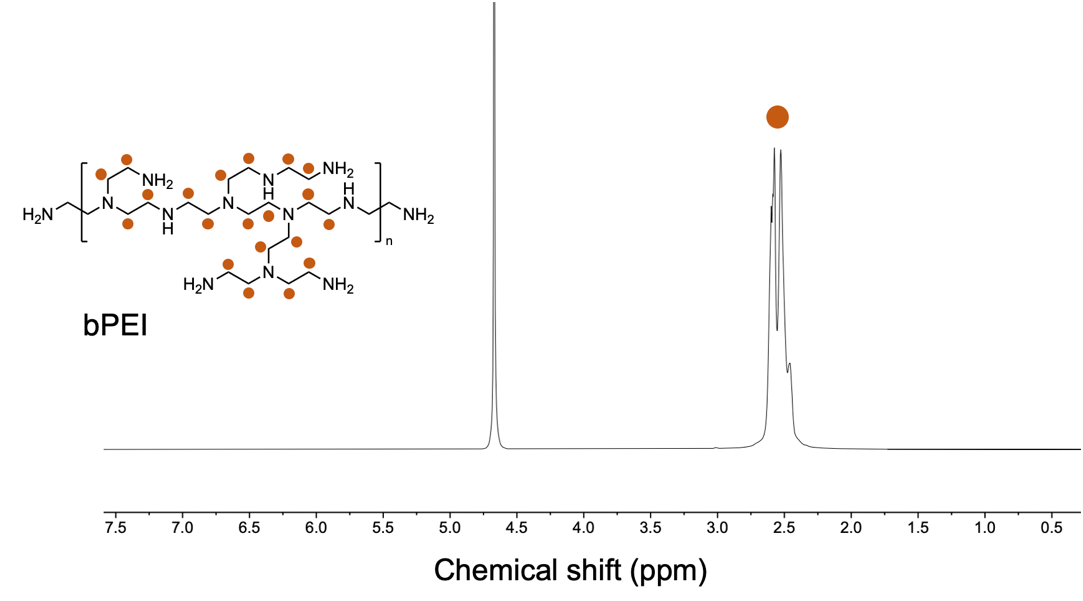
*

**Figure S2** ^1^H-NMR spectrum of bPEI.


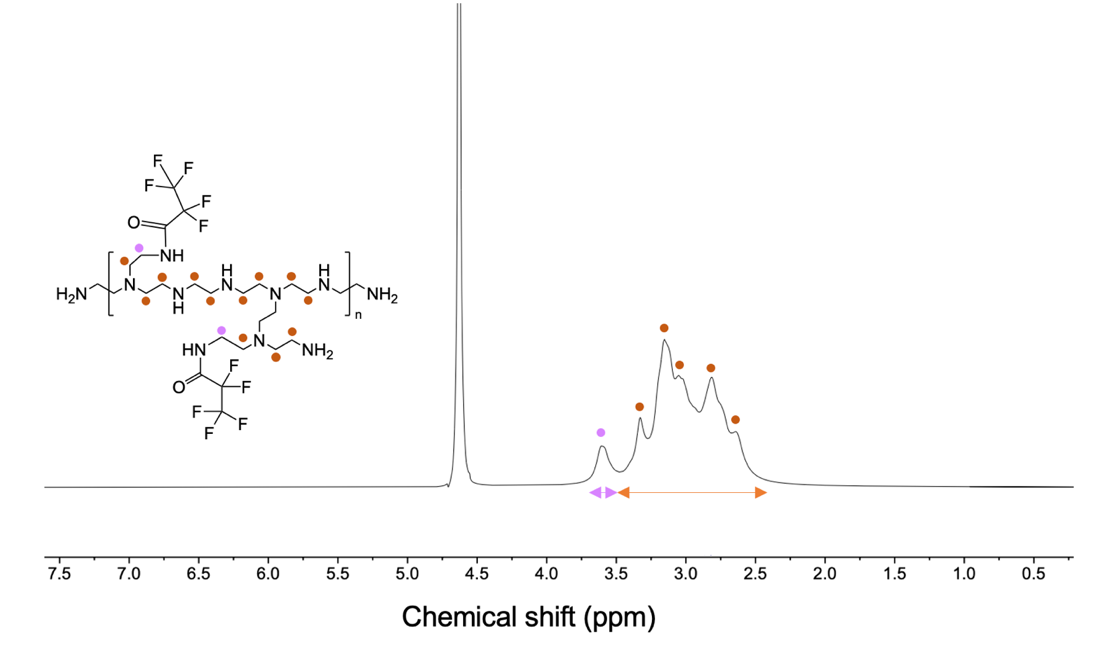


**Figure S3** ^1^H-NMR spectrum of the synthesized PF.

*
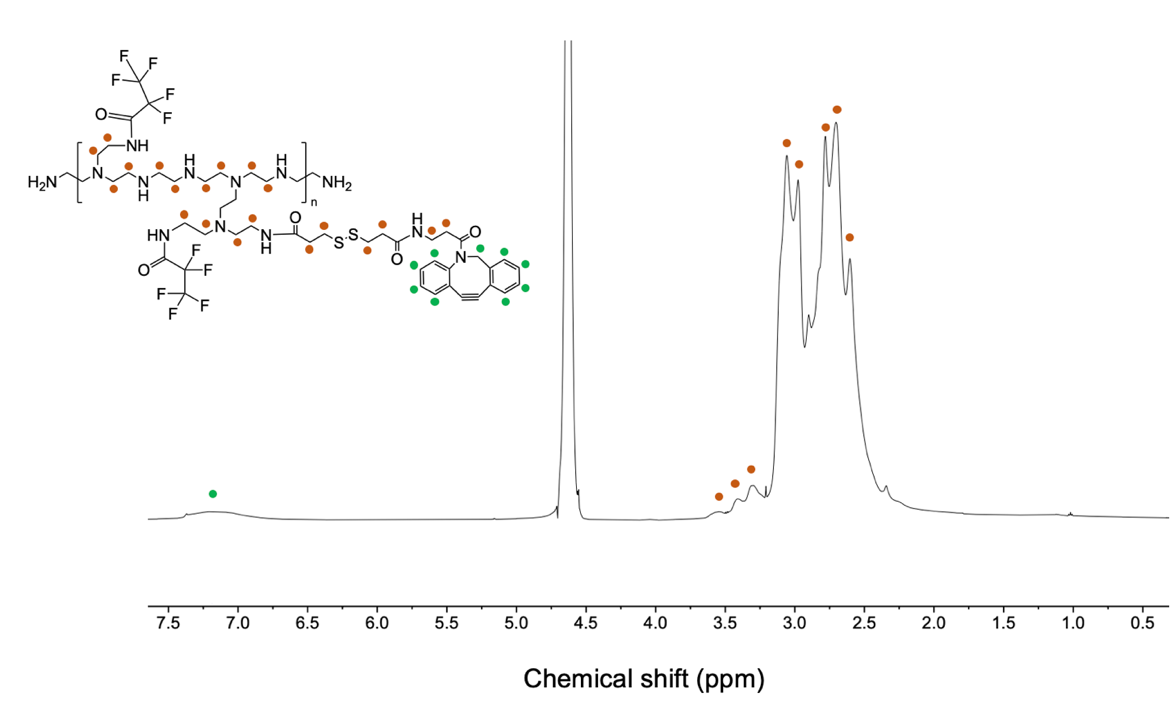
*

**Figure S4** ^1^H-NMR spectrum of the synthesized PF-DBCO.

*
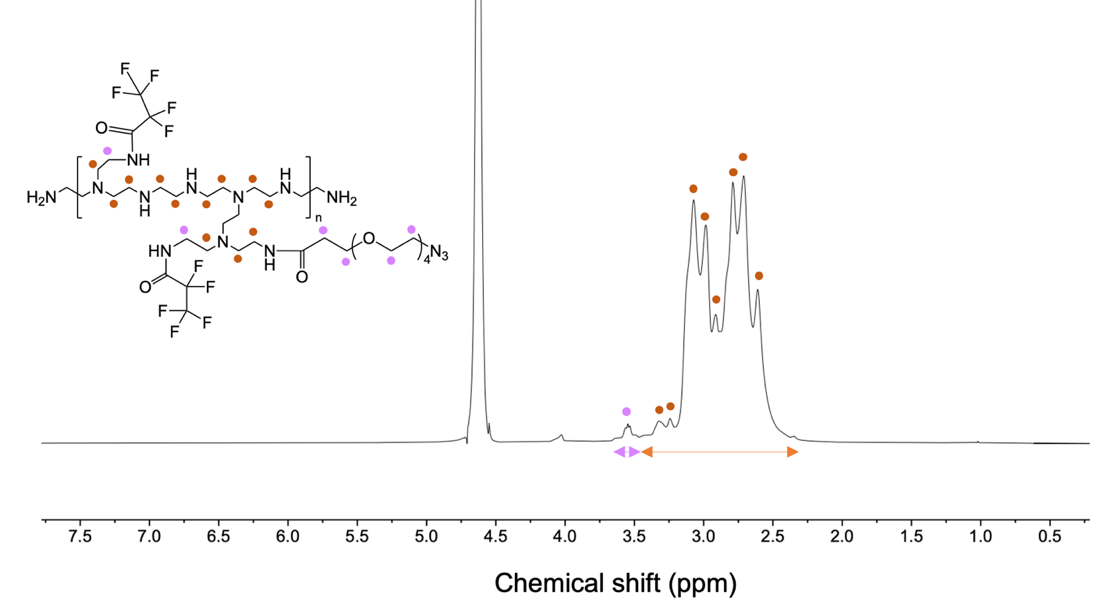
*

**Figure S5** ^1^H-NMR spectrum of the synthesized PF-N_3_.

*
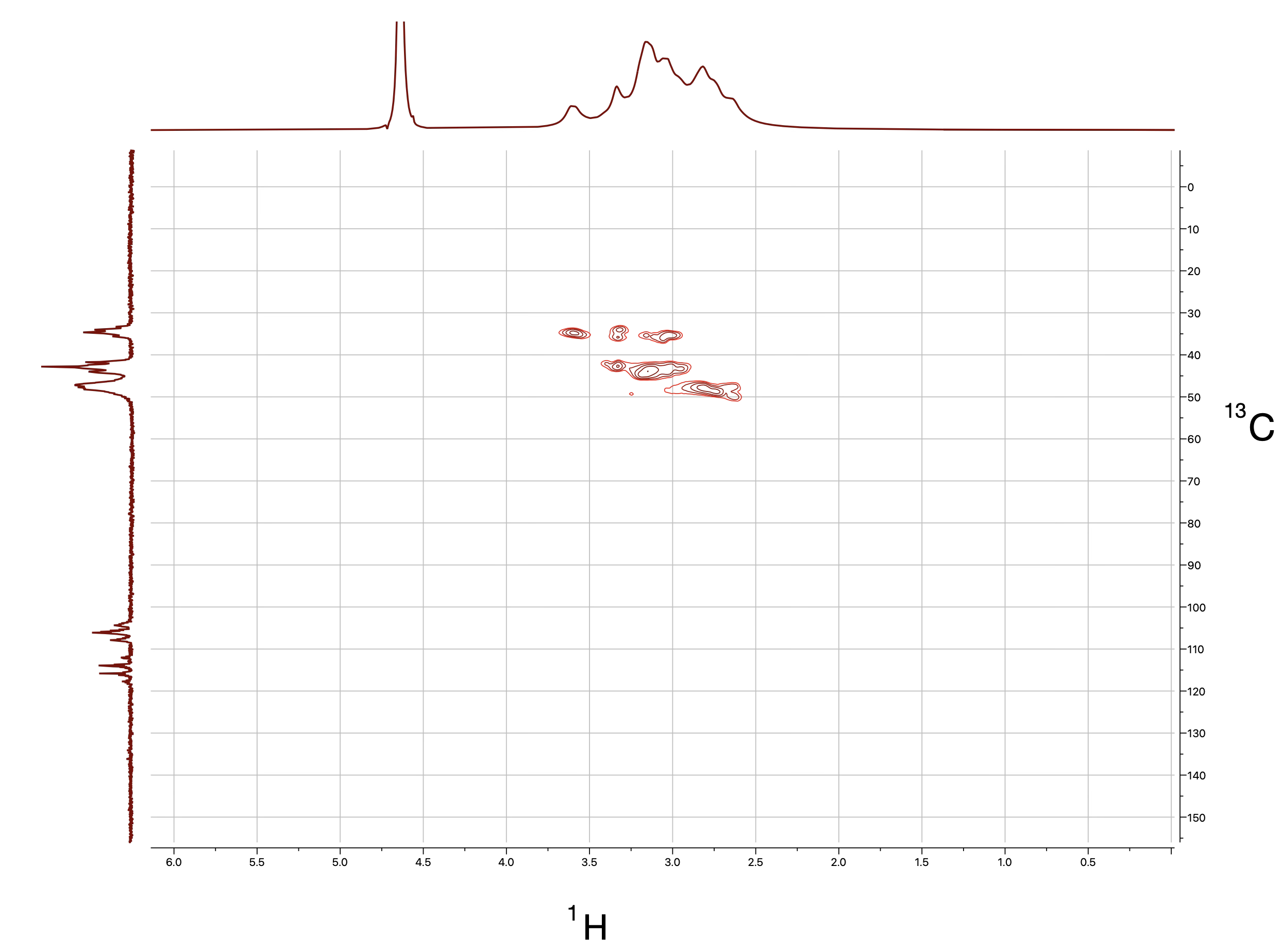
*

**Figure S6** 2D NMR spectroscopy with specifically heteronuclear single quantum coherence (HSQC) for the synthesized PEI-F.

**Table S1.** Assigned HSQC Cross-Peaks and Structural Correlations

| **Peak ID** | **δH (ppm)** | **δC (ppm)** | **Structural Assignment** | **Chemical Environment** | **Substitution Status** |
| --- | --- | --- | --- | --- | --- |
| A | Not applicable | 100–120 | -CF₂- or -CF₃ | Terminal perfluorinated alkyl group | Fluoroalkyl-substituted |
| B | 2.6–3.1 | 45–50 | -CH₂-N< or -CH₂-NH- (inner) | Methylene adjacent to central nitrogen | Unsubstituted backbone |
| C | 2.8–3.5 | 40–45 | -CH₂-NH- (middle) | Middle methylene in ethyleneamine unit | Unsubstituted backbone |
| D | 2.9–3.7 | 30–40 | -CH₂-NH₂ or -CH₂-NH-(F) | Terminal primary amine methylene or fluorine-adjacent methylene | Partially substituted |


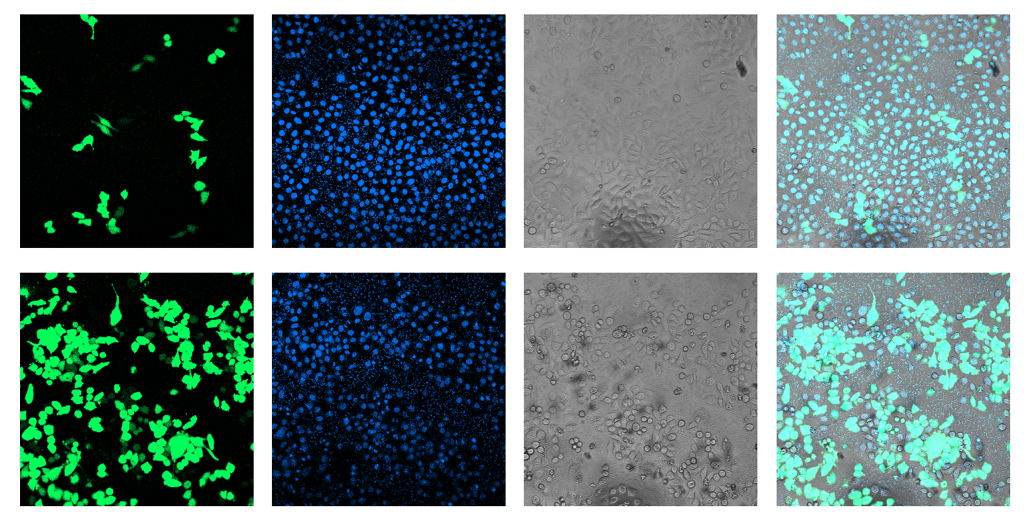


**Figure S7** Transfection of mGFP-encapsulating delivery constructs of Lipofectamine^TM^ 3000 (upper) and PEI-F(N_3_&DBCO) (lower) in U87 cells.


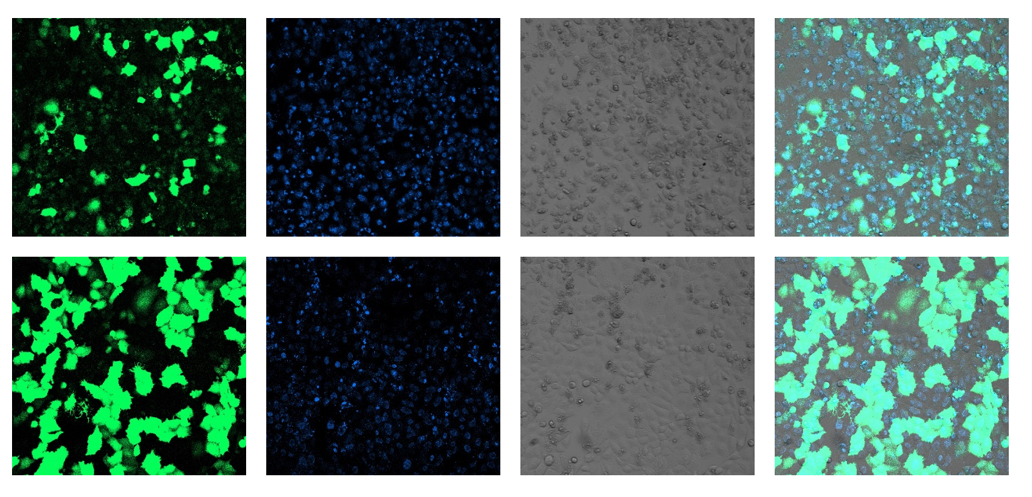


**Figure S8** Transfection of mGFP-encapsulating delivery constructs of Lipofectamine^TM^ 3000 (upper) and PEI-F(N_3_&DBCO) (lower) in Huh-7 cells.


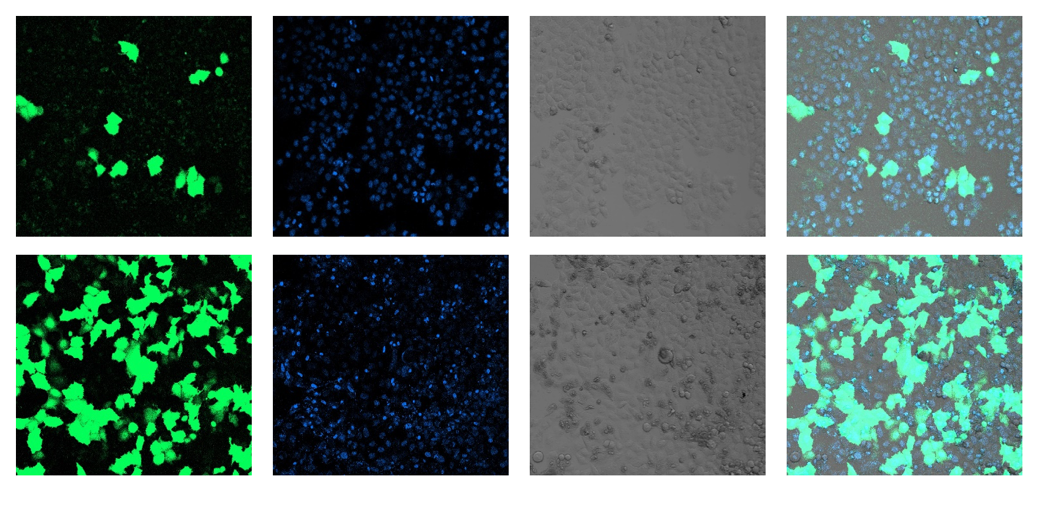


**Figure S9** Transfection of mGFP-encapsulating delivery constructs of Lipofectamine^TM^ 3000 (upper) and PEI-F(N_3_&DBCO) (lower) in PC12 cells.


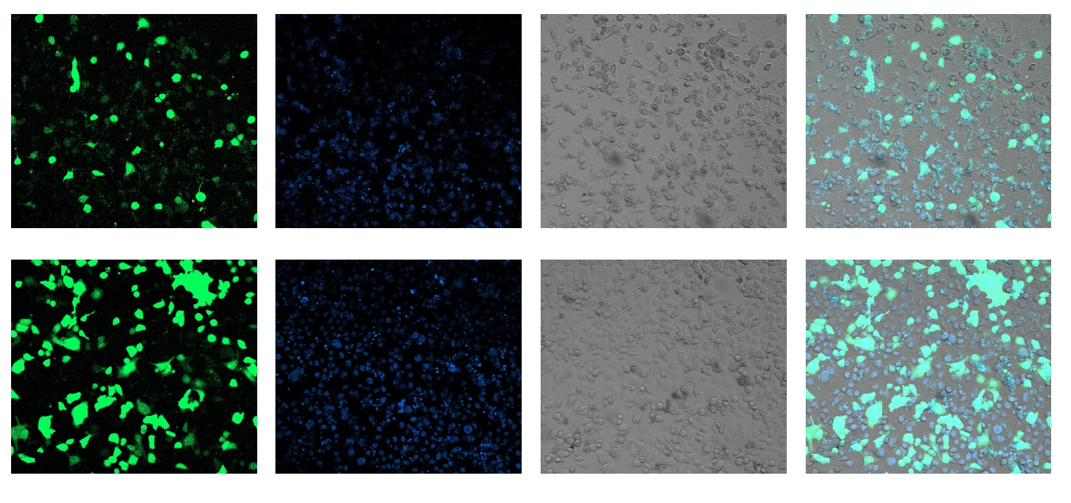


**Figure S10** Transfection of mGFP-encapsulating delivery constructs of Lipofectamine^TM^ 3000 (upper) and PEI-F(N_3_&DBCO) (lower) in RAW264.7cells.


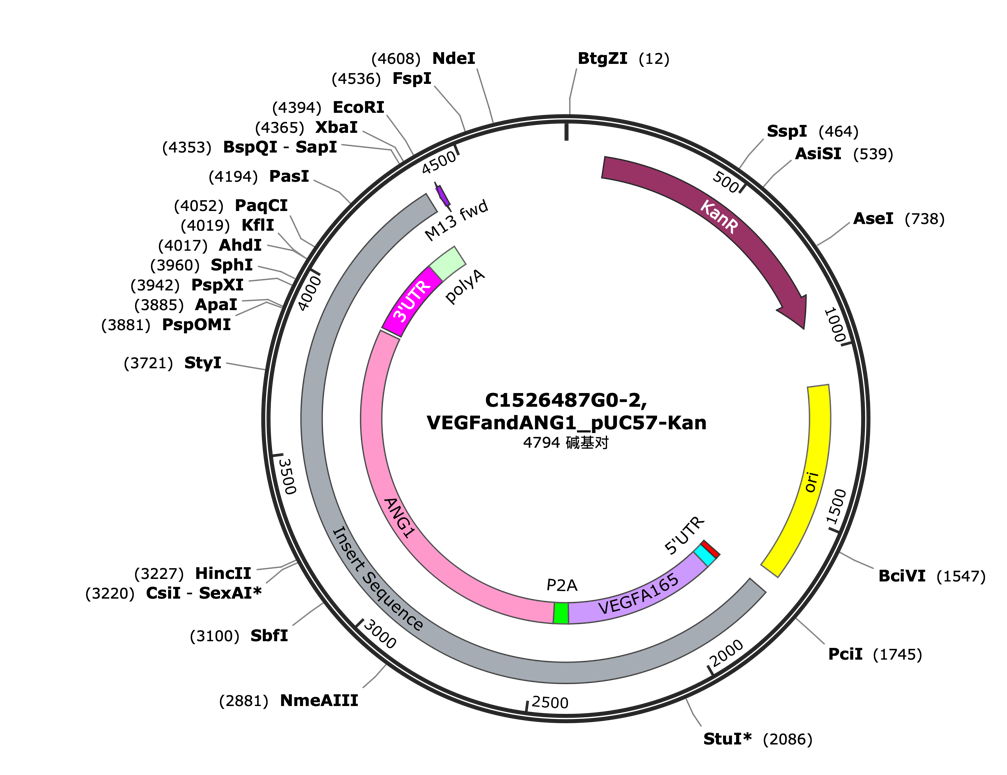


**Figure S11** The constructed pDNA map for subsequent IVT synthesis of VEGF mRNA.

**The T7 Promoter Position and Transcription Start Site.** The GenBank file (pUC57-VEGF-Kan.gb, provided as the enclosed supporting information in the revised submission) confirms the presence of a T7 promoter driving in vitro transcription (Figure S11):

| **Feature** | **Position** | **Sequence** |
| --- | --- | --- |
| T7 promoter core (-17 to -1) | bp 1752–1767 | `5'-AATACGACTCACTATA-3'` |
| Extended T7 context | bp 1751–1767 | `5'-TAATACGACTCACTATA-3'` |
| Transcription start site (+1, G) | bp 1778-69 | `AG` |
| +2 to +6 sequence | bp 1770–1774 | `GTAGT` |


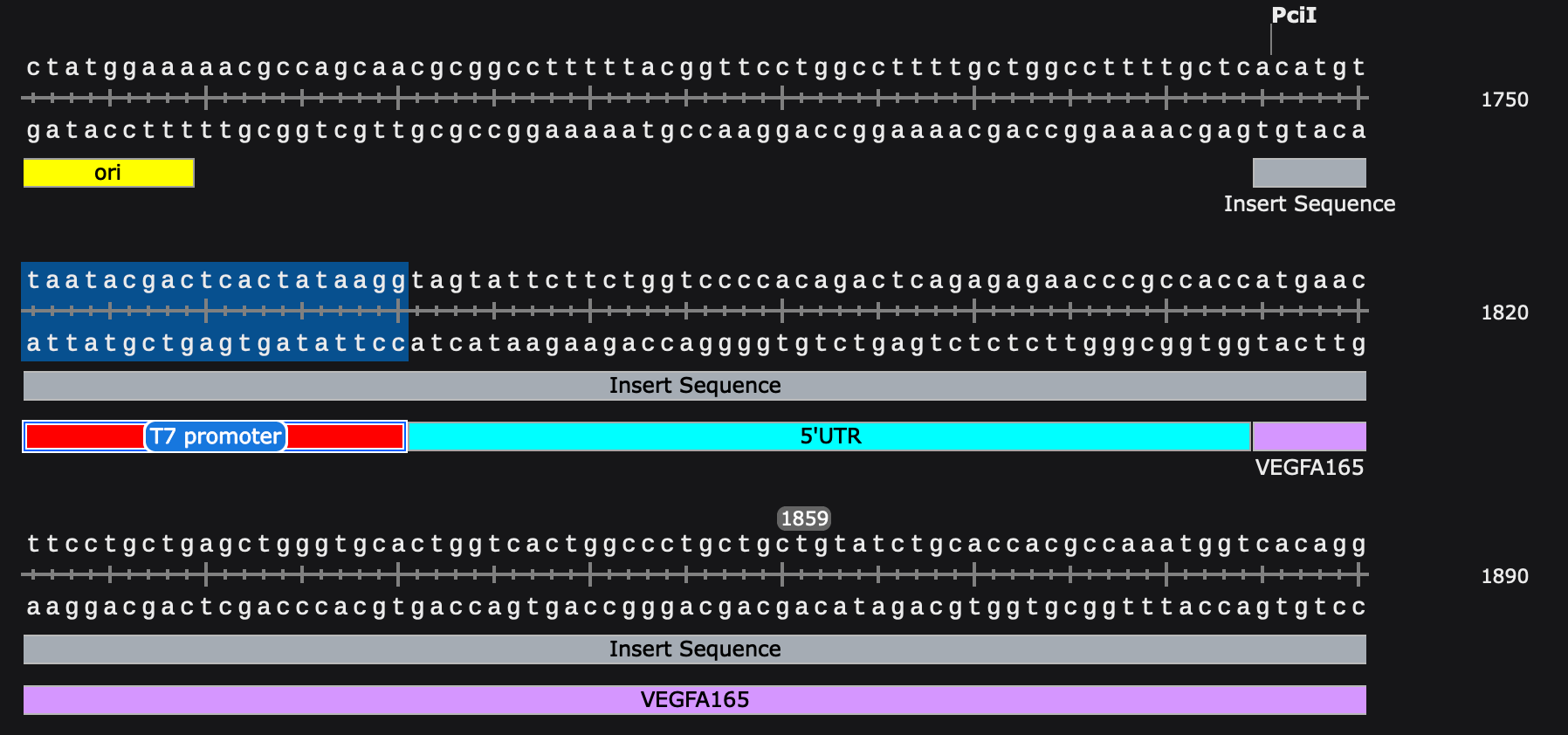


**The Insert Sequence and Its Position.**

| **Feature** | **Details** |
| --- | --- |
| Insert designation | "Insert Sequence" (misc_feature, bp 1745–4370) |
| Insert length | 2,626 bp |
| Insert identity | VEGF-A165–ANG1 fusion cassette |
| 5' end of insert | Immediately downstream of T7 promoter |
| 3' restriction sites within/after insert | PspXI (`ACTCGAGC`, bp 3941–3948); XbaI (`TCTAGA`, bp 4365–4370) |

**Linearization Strategy for In Vitro Transcription.**

| **Parameter** | **Detail** |
| --- | --- |
| Linearization enzyme | XbaI (`TCTAGA`, bp 4365–4370) |
| Purpose | Generate a run-off transcription template |
| Critical requirement | Complete linearization verified by gel electrophoresis |
| Purification | Phenol-chloroform extraction or column purification to remove enzyme and buffer components |


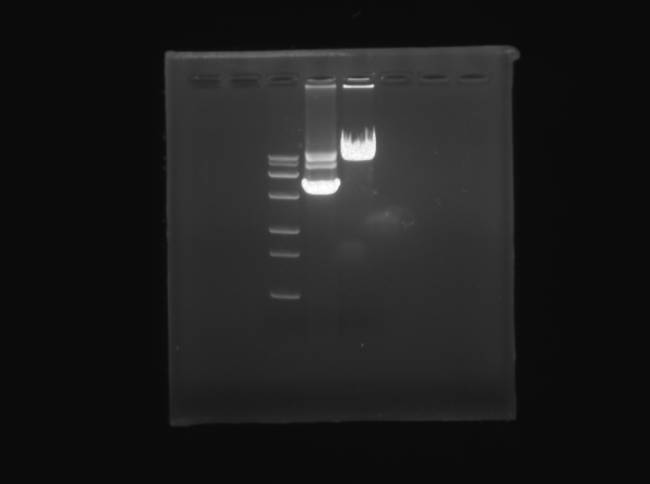


**Figure S12** The constructed pDNA and the linearized form by gel electrophoresis.

**Complete Plasmid Map Description.** The plasmid VEGFandANG1_pUC57-Kan (4,794 bp circular DNA) has the following annotated features:

| **Feature** | **Position** | **Label/Note** |
| --- | --- | --- |
| KanR (aph(3')-Ia) | 114–923 | Kanamycin resistance; confers G418 resistance in eukaryotes |
| ori | 1101–1689 | High-copy-number ColE1/pMB1/pBR322/pUC origin |
| T7 promoter | 1752–1774 | Drives IVT; initiates at G1771 |
| Insert (VEGF-A165–ANG1) | 1745–4370 | "Insert Sequence" |
| M13 fwd primer | complement(4401–4416) | Sequencing primer |

**Protocol for mRNA synthesis from plasmid.**

The plasmid was linearized with XbaI (New England Biolabs) at 37°C for 2–4 hours. Complete linearization was verified by agarose gel electrophoresis (Figure S12). The linearized DNA was purified by phenol-chloroform extraction and ethanol precipitation, then resuspended in nuclease-free water.

Step 1: Template linearization

The plasmid was linearized with XbaI (New England Biolabs) at 37°C for 2–4 hours. Complete linearization was verified by agarose gel electrophoresis (Figure S12). The linearized DNA was purified by phenol-chloroform extraction and ethanol precipitation, then resuspended in nuclease-free water.

Step 2: In vitro transcription with co-transcriptional capping

In vitro transcription was performed using Hi-T7 RNA polymerase (New England Biolabs) with CleanCap AG (Trilink Biotechnologies) for co-transcriptional capping. The 50 μL reaction contained 1–2 μg linearized template, 7.5 mM each ATP, CTP, and UTP, 1.5 mM GTP, 4 mM CleanCap AG, 1× T7 reaction buffer, 1 U/μL murine RNase inhibitor, and 50–100 U Hi-T7 RNA polymerase. The reaction was incubated at 37°C for 2–4 hours.

Step 3: DNase I treatment

The DNA template was degraded by addition of 1 U RNase-free DNase I per μg template DNA and incubation at 37°C for 30 minutes.

Step 4: Purification and quality control

The capped and polyadenylated mRNA was purified by oligo(dT) magnetic beads. Quality was assessed by capillary electrophoresis for integrity and UV spectrophotometry for purity (A260/A280 = 1.8–2.1).
